# Derivation of Equine Mesenchymal Stem/Stromal Cells from Induced Pluripotent Stem Cells via the Neural Crest Pathway and Characterisation by Immunophenotype and Tri-Lineage Differentiation

**DOI:** 10.3390/ani16111618

**Published:** 2026-05-26

**Authors:** Elvira Bernad, Belén Serrano, Arantza Vitoria, Sara Fuente, Antonio Romero, Francisco José Vázquez, Pilar Zaragoza, Clementina Rodellar, Alina Cequier, Laura Barrachina

**Affiliations:** 1 Laboratorio de Genética Bioquímica-LAGENBIO (Universidad de Zaragoza), Instituto Agroalimentario de Aragón-IA2 (Universidad de Zaragoza-CITA), Instituto de Investigación Sanitaria de Aragón-IISA, 50013 Zaragoza, Spain; e.bernad@unizar.es (E.B.); belensepa@unizar.es (B.S.); avm@unizar.es (A.V.); sfuente@unizar.es (S.F.); aromerol@unizar.es (A.R.); pvazquez@unizar.es (F.J.V.); pilarzar@unizar.es (P.Z.); rodellar@unizar.es (C.R.); lbarrach@unizar.es (L.B.); 2 Servicio de Cirugía y Medicina Equina, Hospital Veterinario, Universidad de Zaragoza, 50013 Zaragoza, Spain

**Keywords:** horse, iPSC, MSC, neural crest, differentiation, pluripotency, gene expression

## Abstract

Mesenchymal stem/stromal cells (MSCs) hold promise for treating different equine conditions but enter senescence during culture. Using induced pluripotent stem cells (iPSCs) to derive MSC-like cells (iMSCs) can increase cell availability and diminish the need for invasive and repeated tissue harvesting. While human iMSCs are intensively studied, research on equine iMSCs (eqiMSCs) is very limited and has focused on strategies for spontaneous differentiation to obtain these cells. The aim of this study was to obtain MSC-like cells from equine iPSCs (eqiPSCs) by directing their differentiation via the neural crest pathway. The resulting eqiMSCs downregulated pluripotent gene expression compared to originating eqiPSCs, and the majority of lines met most of the standard criteria for tissue-derived MSCs (immunophenotype and tri-lineage differentiation potential). Nevertheless, eqiMSCs showed some differences from primary equine MSCs, possibly due to their different developmental origin, and displayed certain inter-line variability, which might be related to the different kinetics of independent eqiPSC lines. This study demonstrates for the first time that equine MSC-like cells (eqiMSCs) can be derived from eqiPSCs by directing their differentiation through the neural crest pathway. This constitutes an important advancement towards more sustainable sources of therapeutic cells in veterinary medicine and warrants further exploration of the functional characteristics of these novel cells.

## Simple Summary

Horses often suffer from injuries in tendons and joints that heal slowly. While mesenchymal stem/stromal cells (MSCs), a type of adult stem cell, are a promising treatment, obtaining them from tissues like bone marrow requires invasive procedures that yield limited cell numbers. To solve this, ordinary cells can be “reprogrammed” in the lab to become stem cells with superior differentiation capacity, termed iPSCs. These iPSCs can subsequently differentiate into cells similar to MSCs, termed iMSCs. This is a promising alternative source of cells for therapy, but its development is limited in equine medicine. In this study, we developed a new method to turn these versatile iPSCs into iMSCs, following a pathway similar to natural embryo development. We created several lines of these new iMSCs and confirmed that the majority of them became specialised cells that met most of the standard identity criteria. However, we found that iMSCs also showed some differences compared to the traditional MSCs. This study is the first to prove that equine iMSCs can be obtained by this pathway, representing a significant step forward in veterinary medicine, as it can provide a more sustainable source of cells for future treatments.

## 1. Introduction

Horses frequently suffer from tendon and joint injuries [1,2] and these tissues present poor repair capacity [3]. Thus, regenerative medicine has been attracting growing attention, as it aims to improve tissue repair by using the organism’s own mechanisms. Specifically, mesenchymal stem/stromal cells (MSCs) are very promising for this purpose [4]. The survival of MSCs after transplantation and their engraftment in the target tissue are often poor. Therefore, mounting evidence suggests that MSCs exert their therapeutic effect predominantly by secreting bioactive factors. Such factors could modulate the immune response, reduce inflammation, inhibit cell death, and stimulate endogenous repair processes [3]. These properties equip MSCs for a wide range of therapeutic applications, also beyond musculoskeletal injuries, in human and veterinary medicine. However, MSCs enter senescence after several subculture passages, so they cannot be expanded indefinitely [5], which also hinders the quality of the cell product. This fact, added to the shortage of MSCs found in adult tissues, leads to the need for repeatedly harvesting tissue, which in some cases entails invasive procedures (e.g., bone marrow aspiration). Furthermore, MSCs isolated from different tissue harvests can present differences, which potentially increases the heterogeneity of the cell product [6]. In addition, the number of MSCs obtained tends to decrease with age, usually resulting in fewer cells of poorer quality in elderly animals [7,8]. Hence, it is essential to develop methods that provide a stable supply of therapeutic cells [9].

Induced pluripotent stem cells (iPSCs) constitute an attractive alternative source of cells for therapy. Induced pluripotent stem cells are characterised by their virtually unlimited self-renewing capacity and their ability to generate any cell lineage of an adult organism, which holds great potential for both clinical applications and disease modelling strategies. However, while important advancements have been made for the application of human iPSCs and their derivatives, the veterinary iPSC field remains underdeveloped [10]. This field represents a promising future venue for veterinary medicine, where horses are of particular relevance due to their musculoskeletal physiopathology being similar to humans [11]. However, direct administration of undifferentiated iPSCs is discouraged because of the risks of tumorigenicity and ectopic tissue formation [12]. Instead, these cells can theoretically serve as a source of unlimited numbers of any cell type from any germ layer [13], including other progenitor cells like MSCs. This strategy can increase cell availability, avoid the donor’s age-associated decline in MSC properties, and diminish the need for invasive and repeated tissue harvesting [14].

Therefore, deriving MSC-like cells from iPSCs (termed iMSCs) could incorporate the benefits while overcoming the limitations of both iPSCs and primary MSCs, making iMSCs a promising new cell type for regenerative medicine [15]. This approach is well-established in human medicine [16], where, indeed, three clinical trials are currently underway to evaluate these cells [17]. Such advancements underscore the need to bridge the gap in veterinary medicine, where research into equine iMSCs (eqiMSCs) remains limited. Specifically, only two works have reported the derivation of eqiMSCs to date [15,18]. Due to the limited number of studies, the variability in the methods used, and the limited characterisation of the cells obtained, no standard protocols are currently available to derive eqiMSCs.

Methods for generating MSC-like cells from iPSCs can be broadly classified into two categories: spontaneous differentiation and directed differentiation through defined developmental pathways. Spontaneous differentiation represents a relatively simple approach, but the process is less controllable and often results in more heterogeneous populations. On the other hand, directed differentiation typically requires more complex protocols but is usually more efficient [19]. Directed approaches include iMSC derivation through mesodermal pathways, such as the lateral plate mesoderm (LPM), which reflects the developmental origin of most bone marrow-derived MSCs [20,21,22]. However, a subset of MSC populations in the body arises from the ectoderm via the neural crest, a transient population of multipotent cells that contribute to craniofacial skeletal tissues. Some examples of neural crest-derived mesenchymal cells include those associated with dental structures and peripheral nerves. These cells pose some distinct biological features compared to mesoderm-derived MSCs, including superior proliferation and immunomodulation potential, but they also present more limited formation of hyaline cartilage [23]. The derivation of MSC-like cells through the neural crest pathway has been replicated in vitro from human iPSCs, representing a promising approach for iMSC derivation [9,24,25,26].

Given the important potential advantages of iMSCs for veterinary applications, but the still limited evidence on the generation of these cells in the equine species, the current study aimed to gain insight into this alternative source of cells for therapy. To do that, we explored a directed differentiation method via the neural crest pathway to generate equine MSC-like cells (eqiMSCs) from equine iPSCs (eqiPSCs) by adapting a protocol from the human species. The obtained cells were subjected to formal characterisation following the standard criteria for surface marker expression and tri-lineage differentiation potential, using primary equine MSCs as internal control.

## 2. Materials and Methods

### 2.1. Study Design

Four different lines of eqiPSCs obtained and characterised in a previous study [27] were subjected to a directed differentiation protocol via the neural crest pathway to obtain equine MSC-like cells (eqiMSCs). This protocol consisted of two stages: first, to obtain induced neural crest cells (eqiNCCs), and second, to differentiate them into eqiMSCs. Four lines of eqiPSCs (named FD6, FD7, FD8.1 and FD8.6) were subjected to this protocol at the same time. On the other hand, equine bone marrow-derived MSCs (eqBM-MSCs) from three different donors (D1, D2 and D3), available at our cell bank and used in previous studies [28,29,30], were used as a reference, given their extensive study in the horse [31,32].

**Figure 1:**
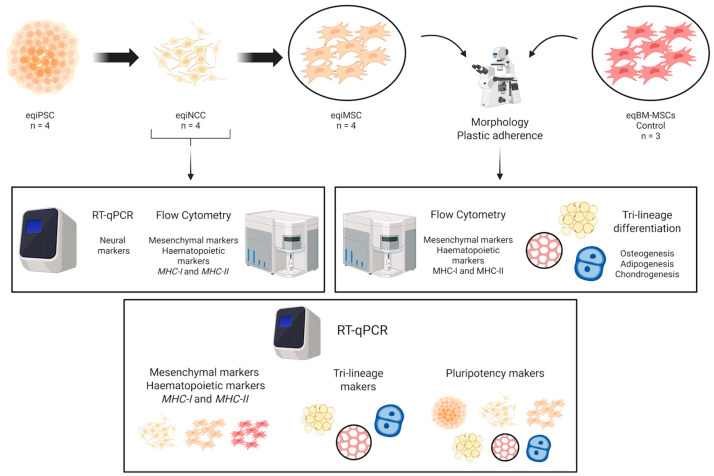
Study design showing the work flow followed. Equine induced pluripotent stem cells (eqiPSCs, *n* = 4) were derived into equine induced neural crest cells (eqiNCCs, *n* = 4), whose identity was checked by gene expression of neural markers and their surface expression of mesenchymal, haematopoietic and immunogenic markers (major histocompatibility complex [MHC] class I and II) was analysed by flow cytometry. Equine iNCCs were subsequently derived into equine mesenchymal stem/stromal cells-like (eqiMSCs, *n* = 4). After checking cell morphology and plastic adherence, eqiMSCs were characterised by analysis of surface markers by flow cytometry and tri-lineage differentiation capacity. Equine bone marrow-derived mesenchymal stem/stromal cells (eqBM-MSCs, *n* = 3) were used in the same assays as a control. Surface markers were also assessed by real-time quantitative polymerase chain reaction (RT-qPCR) in eqiNCCs, eqiMSCs and eqBM-MSCs. Adipogenesis, osteogenesis and chondrogenesis lineage markers were assessed in the adipocytes, osteocytes and chondrocytes derived from eqiMSCs. Pluripotency markers were analysed in eqiPSCs, eqiNCCs, eqiMSCs, and in the adipocytes, osteocytes and chondrocytes differentiated from eqiMSCs.

The four eqiMSC lines obtained, along with the three eqBM-MSC lines used as reference, were subjected to formal characterisation. This included the assessment of mesenchymal, hematopoietic and immunogenic surface markers—using both flow cytometry and real-time quantitative polymerase chain reaction (RT-qPCR)—and of their tri-lineage differentiation potential. In addition, the intermediate population of iNCCs was also analysed for the same set of surface markers to study their evolution along differentiation. Moreover, iNCC identity was confirmed by the expression of genes related to neural stem cells. Finally, the gene expression of pluripotent markers was assessed in all the cell types obtained, from eqiPSCs to eqiNCCs, eqiMSCs, adipocytes, osteocytes and chondrocytes, to provide insight into the evolution of pluripotency along the entire differentiation process. A summary of the study design is represented in Figure 1.

The number of eqiPSC lines used to generate eqiMSCs (*n* = 4) was chosen based on the bibliography [9], and the eqiPSC lines were selected based on characterisation records from a previous study [27] (see Section 2.3). The number of eqiMSC lines characterised (*n* = 4) is consistent with the range typically used in similar studies (usually *n* = 3) [33,34].

### 2.2. Equine BM-MSCs Obtainment and Culture

Bone marrow-derived MSCs from three different donors (namely D1, D2 and D3) were selected from those already available in our cell bank and used in previous studies [28]. The three eqBM-MSC lines were obtained as previously described by our group [35]. In brief, bone marrow was harvested from the sternum of the horses under sedation (0.04 mg/kg IV romifidine, Sedivet, Boehringer Ingelheim, Barcelona, Spain; and 0.02 mg/kg IV butorphanol, Torbugesic, Pfizer, Madrid, Spain) with local analgesia using lidocaine (Anesvet, Laboratorios Ovejero, León, Spain). Mononuclear cells were separated by density gradient centrifugation and seeded in culture medium consisting of low-glucose Dulbecco’s modified Eagle medium (DMEM) supplemented with 10% foetal bovine serum (FBS), 0.1 mg/mL L-glutamine, 100 U/mL penicillin and 100 µg/mL streptomycin (all from Sigma–Aldrich, Madrid, Spain). The cells were expanded until passage three at 37 °C, 5% CO_2_ and 90% relative humidity. The passages were performed by enzymatic digestion with Trypsin-EDTA (0.25%) (Gibco™, Thermo Fisher, Madrid, Spain) and the cells were cryopreserved in freezing medium consisting of 90% FBS and 10% dimethyl sulfoxide (DMSO) (Sigma–Aldrich). The eqBM-MSCs were used between passages 3 and 4.

### 2.3. Equine iPSC Generation and Culture

Four monoclonal lines of eqiPSCs (namely FD6, FD7, FD8.1 and FD8.6) were selected from those generated and characterised in a previous study from our group [27]. Briefly, equine embryo-derived MSCs obtained as in Tasma et al., (2022) [36] were reprogrammed with a lentiviral vector that included four human pluripotency factors (Oct-3/4, Sox2, c-Myc, and Klf4) under the control of the same promoter (CRE-excisable OSKM lentifect purified particles, Cat No. LP801-100, GeneCopoieia, Labomics, Rockville, MD, USA). The four lines were obtained under the same conditions in a single reprogramming experiment using cells from one donor. Each line was monoclonally expanded from individually picked colonies. We selected these four lines as they had been characterised in detail in a previous study and showed a similar profile. These four lines met the standard pluripotency criteria at three levels: cellular (morphology), molecular (expression of pluripotent markers, both gene expression and immunofluorescence) and functional (three germ-layer differentiation capacity, as determined by embryoid body assays in vitro). In addition, a normal karyotype was confirmed in these lines [27].

Equine iPSCs were grown in DMEM KnockOut™ (Gibco™, Thermo Fisher) supplemented with 20% KnockOut Serum Replacement (KOSR; Gibco™, Thermo Fisher), 2 mM L-glutamine (Sigma–Aldrich), 0.1 mM MEM non-essential amino acids solution 100X (NEAA; Gibco™, Thermo Fisher), 0.1 mM 2-mercaptoethanol (Sigma–Aldrich), 1000 U/mL human leukaemia inhibitory factor (LIF; Quimigen, MedChemExpress, Madrid, Spain) and 10 ng/mL human basic fibroblast growth factor (bFGF 154a.a, Quimigen, MedChemExpress). Equine iPSCs were cultured in 6-well plates with 200,000 irradiated mouse embryonic fibroblasts (iMEFs; Gibco™, Thermo Fisher) per well as feeder cells, previously seeded onto Attachment Factor Protein solution (1X) (AF, Gibco™, Thermo Fisher) pre-coated wells, in a humidified incubator at 37 °C, 5% CO_2_ and 90% relative humidity. The culture media were changed every other day, and eqiPSCs were passaged every 5–7 days by enzymatic digestion with StemPro Accutase (Gibco™, Thermo Fisher). The eqiPSCs were used between passages 12 and 16.

### 2.4. Derivation of eqiMSCs from eqiPSCs

A protocol previously described to differentiate human iPSCs into iMSCs via the neural crest pathway [9] was adapted for use in this study in the equine species. This protocol consisted of two stages: first, to differentiate iPSCs into induced neural crest cells (iNCCs), and second, to differentiate those iNCCs into iMSCs. The process was further subdivided into 4 phases: induction of iNCCs from iPSCs, expansion of iNCCs, induction of iMSCs from iNCCs, and expansion of iMSCs.

First, eqiPSCs (*n* = 4) between passages 12 and 16 were cultured in their standard conditions described above in 6-well plates for 4 days (including the use of iMEFs as feeder cells). For eqiNCCs induction, the culture media were directly exchanged (without previously passaging the cells or removing the iMEFs) for iNCC induction media, consisting of a 1:1 mix of Iscove’s Modified Dulbecco’s Medium (IMDM) and Ham’s F12 Nutrient (Gibco™, Thermo Fisher), and supplemented with 1% chemically defined lipid concentrate (CDLC; Gibco™, Thermo Fisher), 1% insulin–transferrin–selenium premix (ITS+ premix; Gibco™, Thermo Fisher), 450 μM 1-thioglycerol (Sigma–Aldrich), 100 U/mL penicillin, 100 µg/mL streptomycin, 10 μM SB431542 and 1 μM CHIR99021 (Quimigen, MedChemExpress) for 10 days. The media were changed every other day during the first 6 days, and every day during the last 4 days. On the 11th day, the cells were passaged with StemPro Accutase (Gibco™, Thermo Fisher) and were considered eqiNCCs at passage 0 (P0).

These eqiNCCs P0 were seeded for expansion at a ratio between 1:1 and 1:3 onto vitronectin-coated wells with the same iNCC induction media but removing CHIR99021 and adding 20 ng/mL bFGF and 20 ng/mL epidermal growth factor (EGF; Quimigen, MedChemExpress). In this second phase, eqiNCCs were expanded under the same conditions until passage 4 (P4). The passages were carried out by enzymatic digestion with StemPro Accutase (Gibco™, Thermo Fisher) when the cells reached 80–90% confluence or at day 7 at the latest to prevent excessive vitronectin degradation.

Twenty-four hours after plating P4 eqiNCCs onto vitronectin, the medium was replaced with the MSC growth medium described above for eqBM-MSCs and supplemented with 20 ng/mL of bFGF to initiate the induction of eqiMSCs. At this point, the cells were considered eqiMSCs at P0. This third phase lasted for 14 days, during which cells were passaged when they reached 80–90% confluence. From passage 2 onwards, the substrate (vitronectin) was removed, and the cells were directly seeded onto uncoated tissue-treated culture plates/flasks.

This point is considered the beginning of the fourth and last phase: the expansion of iMSCs, whose hallmark is the progressive stabilisation of the cell populations. This phase was conducted under the same conditions used for eqBM-MSC expansion but initially maintaining bFGF in the media. Basic FGF was gradually withdrawn from the culture medium by dividing in half the concentration in each subsequent passage, and it was completely removed once the cells exhibited a homogeneous spindle-shaped morphology and sustained growth. The seeding density during the eqiMSC expansion phase was adjusted over time, starting with 10,000 cells/cm^2^ in the first passages and reducing it to 5000–7000 cells/cm^2^ in later passages. When the cells reached passage 6, they were considered stable eqiMSCs based on morphology and growth rate comparable to BM-MSCs at passages 3 and 4, and thus this passage was set for characterisation.

### 2.5. Characterisation of the Obtained eqiMSCs

#### 2.5.1. Immunophenotyping of eqiMSCs

The surface expression of the mesenchymal markers CD44, CD90, CD105, the haematopoietic markers CD45 and CD11α/CD18, and the immunogenic markers MHC-I and MHC-II was analysed by flow cytometry. These markers were selected following the recommendations for reporting animal MSCs [37]. The analysis was conducted in four paired lines of eqiNCCs (FD6, FD7, FD8.1 and FD 8.6; passages 8–10) and eqiMSCs (FD6, FD7, FD8.1 and FD 8.6; passage 6), as well as in three lines of eqBM-MSCs (D1, D2 and D3; passages 3–4).

Each cell type was cultured in their corresponding conditions previously described, and the cells were detached and suspended in phosphate-buffered saline (PBS, Gibco™, Thermo Fisher) at 10^6^ cells/mL. In total, 100 µL of cell suspension per well was placed in a 96-well U-bottom non-treated cell culture plate. Two technical replicates were performed for each staining. The cells were centrifuged at 300 g for 5 min to discard the supernatant and subsequently incubated for 30 min at 4 °C with 50 µL of 1:50 diluted primary antibodies shown in Table 1.

All the antibodies were previously described in the bibliography and/or verified in our lab to label equine cells. Some antibodies were incubated together in the same sample when conjugated to different fluorophores, prioritising combinations of a positive with a negative marker (i.e., CD44 with CD45; CD105 with CD11α/CD18). One replicate of each sample was left unstained to serve as a negative control and to set the gate for positive cells. Following antibody incubation, cells were washed with PBS and stained with 1 µL/well of Ghost Dye Violet 510 (Tonbo Bioscience, Bio-Rad, Barcelona, Spain) for 30 min at 4 °C to discriminate non-viable cells. After completing the staining process, the cellular suspension was transferred into cytometry tubes and at least 10,000 viable events were acquired on a biexponential fluorescence scale in a Gallios flow cytometer (Beckman Coulter, Madrid, Spain). Data were analysed with Kaluza Analyses Version 2.2.1 Software (Beckman Coulter). The gating strategy was previously described by our group [28]. Briefly, dead cells and doublets were excluded, and the positive cell gate was set using unstained controls to record the percentage of positive cells for each marker.

#### 2.5.2. Tri-Lineage Differentiation Assays

Equine iMSCs (passage 6, *n* = 4) and eqBM-MSCs (passage 3, *n* = 3) were subjected to tri-lineage differentiation. Three technical replicates were run for each experimental and control condition. Non-differentiated controls were run using the same seeding density and regular growth media.

##### Osteogenic Differentiation

Cells were seeded at 20,000 cells/cm^2^ in 12-well plates and cultured for 9 days with osteogenic medium. Three different media were tested to induce osteogenesis in eqiMSCs. The first osteogenic medium consisted of MSC growth medium with 5% FBS and supplemented with 10 nM dexamethasone, 10 mM β-glycerophosphate and 100 µM ascorbate-2-phosphate (all from Sigma–Aldrich). This osteogenesis induction medium has been commonly used by our group for eqBM-MSCs [43]. The second osteogenic medium was the same as the first but supplemented with 200 ng/mL Bone Morphogenetic Protein 7 (BMP7; Quimigen, MedChemExpress) [44]. The third osteogenic medium was the same as the first but supplemented with 7.5 mM CaCl_2_ (Sigma–Aldrich) [45]. After 9 days in each osteogenic condition, the cells were fixed in 4% paraformaldehyde (PFA, Thermo Fisher) for 30 min and stained with 2% alizarin red S (Sigma–Aldrich) for 10 min, as previously described [43], to assess changes in morphology and deposition of calcium.

##### Adipogenic Differentiation

The cells were seeded at 5000 cells/cm^2^ in 12-well plates and cultured for 14 days with adipogenic medium, consisting of MSC growth medium supplemented with 1 µM dexamethasone, 500 µM 3-isobutyl-1-methylxanthine (IBMX), 200 µM indomethacin and 15% rabbit serum (all from Sigma–Aldrich). The adipogenic differentiation was examined by fixing the cells in 4% PFA for 15 min, followed by staining with 0.3% oil red O (Sigma–Aldrich) for 30 min at 37 °C [43,46], to assess the formation of lipid vacuoles.

##### Chondrogenic Differentiation

Three hundred thousand cells were pelleted into 15 mL conical polypropylene tubes and cultured for 28 days with chondrogenic medium, consisting of high-glucose DMEM supplemented with 10% FBS, 100 U/mL penicillin and 100 µg/mL streptomycin, 0.1 mg/mL L-glutamine, 1 mM sodium pyruvate (Gibco™, Thermo Fisher), 10 ng/mL transforming growth factor-beta 3 (TGFβ-3; R&D Systems, Barcelona, Spain), 1% ITS+ premix (Gibco™, Thermo Fisher), 40 µg/mL proline (Sigma–Aldrich), 50 µg/mL ascorbate-2-phosphate and 0.1 µM dexamethasone. To assess chondrogenic differentiation, the pellets were fixed in 4% PFA, embedded in paraffin and cut into 5 µm sections. The tissue sections were stained with alcian blue dye to evaluate morphology (e.g., lacunae formation) and proteoglycan deposition [43].

#### 2.5.3. Gene Expression Analysis

Real-time quantitative polymerase chain reaction (RT-qPCR) was used to analyse gene expression in different situations. First, to confirm the identity of eqiNCCs (*n* = 4, passage 8–10) derived from eqiPSCs using the neural crest markers *Ras Homolog Family member B* (*RHOB*) and *Nestin* (*NES*) [47,48]. Second, the expression of eight genes coding for markers commonly used for MSC characterisation was analysed on eqiNCCs (*n* = 4, passage 8–10), eqiMSCs (*n* = 4, passage 6) and eqBM-MSCs (*n* = 3, passage 3). These genes included mesenchymal markers (*CD44*, *CD90*, *CD105*, *CD73*), haematopoietic markers (*CD34*, *CD45*) and immunogenic markers (*MHC-I* and *MHC-II*). Third, RT-qPCR was used to assess lineage-specific markers in the tri-lineage differentiation assay, analysing both undifferentiated and differentiated eqiMSCs. Alkaline phosphatase (*ALP*) was used for osteogenic differentiation (iOM, *n* = 4), peroxisome proliferator-activated receptor γ (*PPARγ*) for adipogenic differentiation (iAM, *n* = 4) and collagen type II (*COL2A1*) for chondrogenic differentiation (iCM, *n* = 4). Fourth, the expression of 5 pluripotent genes (*NANOG*, *POU5F1*, *SOX2*, *FGF5* and *ZFP42*) and the expression of the lentiviral transgene delivered for eqiPSC reprogramming [27] was analysed in eqiPSCs, eqiNCCs and eqiMSCs, as well as in iOM, iAM and iCM (*n* = 4, paired lines).

The primers used for detecting the expression of all these genes were previously reported by our group [27,49]. The primers for *RHOB* and *NES* were designed in this study using the Primer-BLAST online tool of the National Centre for Biotechnology Information (https://www.ncbi.nlm.nih.gov/tools/primer-blast/, accessed on 15 March 2026). The efficiency (E) of the two primer pairs amplifying *RHOB* and *NES* transcripts was 94% and 101%, respectively, as calculated using E = (10^−1^/slope) × 100, where the slope is obtained from a calibration curve performed with 1:5 cDNA serial dilutions. All the primers are presented in Table 2.

##### RNA Extraction and cDNA Reverse Transcription

Each cell type was cultured under their standard conditions described above (eqiPSCs, *n* = 4, passages 14–16; eqiNCCs, *n* = 4, passages 8–10; eqiMSCs, *n* = 4, passage 6; corresponding tri-lineage differentiated cells, *n* = 4 per lineage, differentiated—iOM, iAM, iCM—and non-differentiated; eqBM-MSCs, *n* = 3, passages 3–5), washed with PBS and stored at −80 °C until mRNA was extracted. Isolation of mRNA was performed using the TRIzol^®^ Reagent (15596026, invitrogen, Thermo Fisher) method and the commercial Direct-zol RNA Miniprep Kit (R2052, Zymo Research, Laboaragon, Zaragoza, Spain) according to the manufacturer’s instructions. Prior to reverse transcription, mRNA concentration of each sample was measured with a NanoDrop™ 2000/2000c spectrophotometer (Thermo Fisher), and purity was assessed using 260/280 and 260/230 absorbance ratios. Complementary DNA (cDNA) synthesis was performed from 100 ng of total mRNA per sample using qScript cDNA SuperMix (Quanta Biosciences, Beverly, MA, USA), according to the manufacturer’s instructions. The resulting cDNA was diluted 1:10 in ultrapure nuclease-free distilled water.

##### Real-Time Quantitative PCR Analysis

Gene expression was assessed by RT-qPCR using the Quant Studio 3 Real-Time PCR Instrument (Applied Biosystems, Thermo Fisher). For each sample, 5 µL of Fast SYBR Green Master Mix (Applied Biosystems, Thermo Fisher), 0.15 µL of each primer (300 nM) and 2.7 µL of ultrapure nuclease-free distilled water were used for a final volume of 8 µL, with the addition of 2 µL of cDNA as template, or 2 µL of ultrapure nuclease-free distilled water for the negative (non-template) control (NTC). The amplification reaction was performed in triplicate following these conditions: 20 s at 95 °C for initial activation, followed by 40 cycles consisting of 3 s at 95 °C and 30 s at 60 °C. A dissociation curve protocol was run after every reaction to check for specific amplification. Gene expression was analysed by the comparative 2^−ΔΔCt^ method. The expression of each sample was normalised using the geometric mean of the quantity of two housekeeping genes (*GAPDH*, *glyceraldehyde 3-phosphate dehydrogenase*, and *B2M*, *beta-2 microglobulin*). Reference samples to calculate fold changes for relative quantification were used depending on the group of genes analysed: the line FD8.6 was used for the NCC markers; for the MSC markers and for the pluripotency-associated genes; for the expression of tri-lineage-specific genes, each differentiated eqiMSC line was compared to its respective non-differentiated control.

### 2.6. Statistical Analysis

The software GraphPad Prism 8 (San Diego, CA, USA) was used for data statistical analysis and graphical representation. Data obtained from flow cytometry and RT-qPCR experiments were checked for normality using the Shapiro–Wilk test, and parametric or non-parametric tests were applied accordingly. To compare two independent groups (eqiMSCs and eqBM-MSCs), a non-paired Student’s *t*-test was used when data followed a normal distribution, whereas the Mann–Whitney test was applied for non-normal data. To compare paired samples (eqiNCCs and eqiMSCs), a paired Student’s *t*-test was used for normally distributed data, and the Wilcoxon test for non-normal data. To compare gene expression of pluripotency markers among multiple groups (eqiPSCs, eqiNCCs, eqiMSCs, iOM, iAM, and iCM), Friedman or Kruskal–Wallis tests were used for related or independent samples, respectively, followed by Dunn’s test as a post hoc analysis. Statistical significance was set as *p* < 0.05 in all cases.

## 3. Results

In this study, we established a novel differentiation approach to generate equine MSC-like cells from eqiPSCs (eqiMSCs), based on the adaptation of human iMSC protocols that direct lineage commitment through a neural crest intermediate [9]. This strategy enabled the derivation of eqiMSCs from four out of four independent eqiPSC lines. To our knowledge, this represents the first report of a neural crest-based protocol for eqiMSC generation in the equine species. Notably, prior to implementing this strategy, we evaluated the two protocols previously described in the equine species to derive iMSCs, both based on spontaneous differentiation [15,18]. Additionally, we tested another protocol reported for human cells that was based on directed differentiation through the LPM [22]. However, under our experimental conditions, none of these approaches yielded consistent results (Supplementary Material S1).

### 3.1. Equine MSC-like Cells Could Be Derived from eqiPSCs by Directed Differentiation via the Neural Crest Pathway

**Figure 2:**
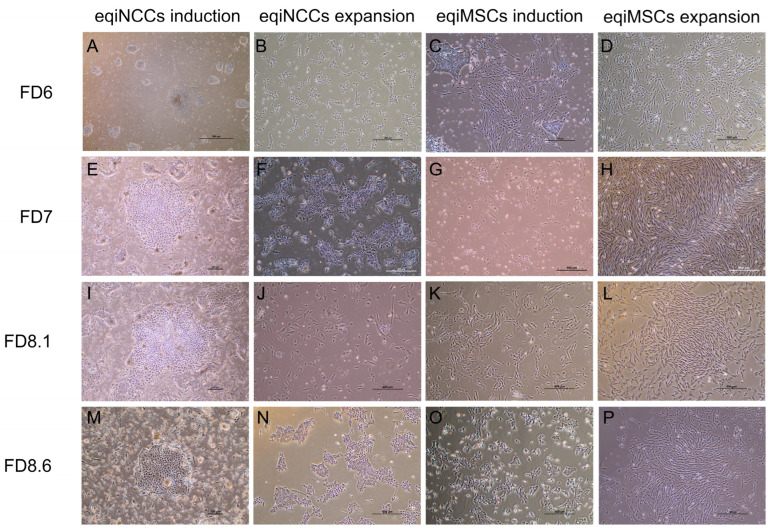
Equine induced mesenchymal stem/stromal cells-like (eqiMSC) derived from equine induced pluripotent stem cells (eqiPSCs) via the neural crest pathway. From left to right columns: equine induced neural crest cells (eqiNCCs) induction, eqiNCCs expansion, eqiMSCs induction and eqiMSC expansion. From top to bottom row: lines FD6, FD7, FD8.1 and FD8.6. Scale bar: (**A**–**D**,**F**–**H**,**J**–**L**,**N**–**P**) = 500 µm (**E**,**I**,**M**) = 100 µm.

The protocol adapted from human literature [9] consisted of using different media over time to provide the cells with the necessary stimuli to differentiate, first into neural crest cells, and secondly into MSC-like cells (Figure 2). Each of these two stages is further subdivided into two phases, induction and expansion, of iNCCs and iMSCs, respectively. In the first phase (iNCC induction), the iMEFs used as a feeder layer gradually died, but the eqiPSCs remained attached, losing their typical high nucleus:cytoplasm ratio and their colony shape with well-defined borders (Figure 2, first column; 2A, 2E, 2I, 2M). Subsequently, during the iNCC expansion phase, the cells acquired a triangle-like shape and expanded around the initial colony (Figure 2, second column; 2B, 2F, 2J, 2N), presenting a high proliferation rate. In the third phase (iMSC induction), the cells acquired the typical MSC-like spindle shape and increased their plastic adherence, but cell clusters remained present (Figure 2, third column; 2C, 2G, 2K, 2O). Along subsequent passages during the fourth phase (iMSC expansion), these cells grew individually, expanding all over the plate (Figure 2, fourth column; 2D, 2H, 2L, 2P), being visually indistinguishable from eqBM-MSCs. The protocol was reproduced under the exact same conditions with four independent eqiPSC lines: FD6 (Figure 2, first row; 2A, 2B, 2C, 2D), FD7 (Figure 2, second row; 2E, 2F, 2G, 2H), FD8.1 (Figure 2, third row; 2I, 2J, 2K, 2L) and FD8.6 (Figure 2, fourth row; 2M, 2N, 2O, 2P). Morphological changes along the differentiation process occurred at different paces, with FD8.1 exhibiting a more mature phenotype at earlier stages, whereas FD8.6 showed a slower progression.

### 3.2. Expression of Neural Crest Markers Confirms Identity of Equine iNCCs

**Figure 3:**
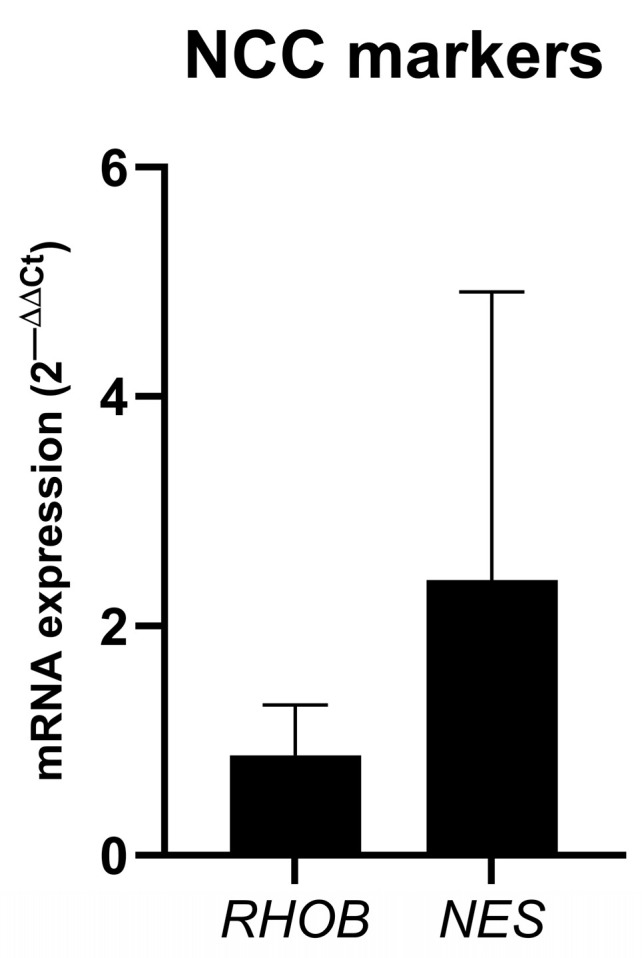
Mean ± S.E.M. of the relative gene expression of the neural crest cell (NCC)-associated markers *Ras Homolog Family Member B* (*RHOB*) and *Nestin* (*NES*) in equine induced neural crest cells (eqiNCCs, *n* = 4). The line FD8.6 was used as a reference sample for normalisation in relative quantification.

The identity of the eqiNCCs, obtained as an intermediate cell population during differentiation, was confirmed by the positive expression of NCC markers (*RHOB* and *NES*) using RT-qPCR. The expression of both *RHOB* and *NES* was observed in all four eqiNCC lines (Figure 3).

### 3.3. Cell Immunophenotype Along the Differentiation and Compared to eqBM-MSCs

**Figure 4:**
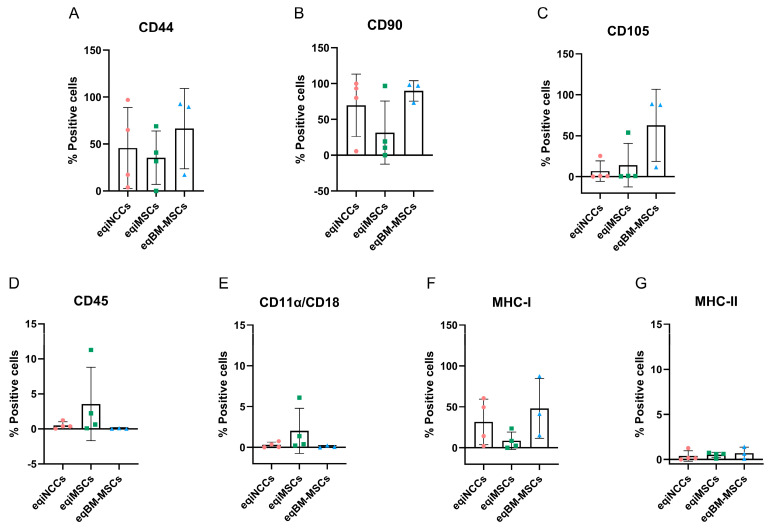
Mean ± S.E.M. of the percentage (%) of positive cells for the surface markers (**A**) CD44 (hyaluronate receptor), (**B**) CD90 (Thy-1), (**C**) CD105 (Endoglin), (**D**) CD45, (**E**) CD11α/CD18, (**F**) MHC-I (Major Histocompatibility Complex type I) and (**G**) MHC-II (Major Histocompatibility Complex type II) analysed in eqiNCCs (equine induced neural crest cells; *n* = 4, pink dots), eqiMSCs (equine induced mesenchymal stem/stromal cells-like; *n* = 4, green dots) and eqBM-MSCs (equine bone marrow-derived mesenchymal stem/stromal cells; *n* = 3, blue dots). No significant differences across cell types were found.

The immunophenotype of eqiNCCs, eqiMSCs and eqBM-MSCs was studied to assess the changes experienced by the cells transitioning from eqiNCCs to eqiMSCs, as well as to detect similarities and differences compared to eqBM-MSCs. Seven surface markers were analysed by flow cytometry, three of which are considered positive markers of equine MSCs (hyaluronate receptor, CD44; Thy-1, CD90; endoglin, CD105), and two markers that are considered negative for equine MSCs (the hematopoietic markers CD45 and CD11α/CD18). In addition, the surface expression of major histocompatibility complex class I (MHC-I) and II (MHC-II) was analysed. Table 3 presents the percentage of positive cells found per marker in each individual line of eqiNCCs, eqiMSCs and eqBM-MSCs. In addition, the mean percentage of positive cells of each type (eqiNCCs, eqiMSCs and eqBM-MSCs) for each marker is presented in Figure 4 to show general trends.

Percentage (%) of positive cells (eqiNCCs: equine induced neural crest cells, eqiMSCs: equine induced mesenchymal stem/stromal cells-like, eqBM-MSCs: equine bone marrow-derived mesenchymal stem/stromal cells) for the surface markers CD44 (hyaluronate receptor), CD90 (Thy-1), CD105 (endoglin), CD45 and CD11α/CD18 (hematopoietic markers), MHC-I and MHC-II (major histocompatibility complex I and II) were determined by flow cytometry.

Statistically significant differences across cell types were not found for any surface marker, likely because of the deviation observed in all cell types. Nevertheless, some distinct patterns were noted. Regarding the markers customarily considered as positive for equine MSCs [37], CD44 was expressed by both eqiMSCs and eqBM-MSCs, but eqiMSCs presented lower percentages of CD44+ cells. Both eqBM-MSCs and eqiMSCs showed inter-donor/line variability. Equine BM-MSCs from one donor (D3) expressed lower levels of CD44 compared to the other donors, but expression was detectable, while one line of eqiMSCs (FD8.6) showed negligible CD44 positivity (0.02%). In the case of eqiNCCs, the mean percentage of CD44+ cells was similar to eqiMSCs and eqBM-MSCs, but there was more variability, with two lines presenting high CD44 levels (FD6 and FD8.1) and two lines presenting considerably lower CD44 levels (FD7 and FD8.6) (Table 3; Figure 4A).

For CD90, 3 out of 3 lines of eqBM-MSCs and 3 out of 4 lines of eqiNCCs showed similarly high percentages of positive cells. However, in the case of eqiMSCs, only one line (FD6) was almost 100% positive, while the other lines showed less than 20% of CD90+ cells, and the line FD8.6 lacked CD90 expression (Table 3; Figure 4B).

The marker CD105 was found positive only in eqBM-MSCs, with a mean percentage of positive cells higher than 60%, yet one line (D3) expressed considerably lower levels of this marker. Only one line of eqiNCCs and one line of eqiMSCs (FD8.1 in both cases) expressed CD105, the latter showing 53.92% of CD105+ cells (Table 3; Figure 4C). Owing to the more variable results of CD90 and CD105 between MSC types and across lines, representative flow plots are presented in Supplementary Material S2 for a more detailed representation of their expression in eqiMSCs.

All cell types were negative for the markers CD45 and CD11α/CD18. However, a small percentage of some eqiMSC lines expressed these markers. Equine iMSCs showed a mean percentage of CD45+ cells lower than 3.55%, with the line FD6 showing higher levels (11.28%, Table 3; Figure 4C). Less than 2% of eqiMSCs were CD11α/CD18+ on average, with FD8.6 showing higher levels (6.08%, Table 3; Figure 4D).

Finally, eqiMSCs expressed lower and more homogeneous levels of MHC-I compared to eqBM-MSCs. The mean percentage of positive eqiMSCs was 8.64%, with the highest expressing line (FD6) showing values below 25%. On the other hand, the mean percentage of positive eqBM-MSCs was 48.8%, with the lowest expressing line (D1) showing values above 15% (Table 3; Figure 4F). In the case of MHC-II, all the eqiMSCs and eqBM-MSC lines were considered negative, with percentages of positive cells below 1.5% in all cases (Table 3; Figure 4G).

In summary, two eqiMSC lines (FD6, FD8.1) were positive for 2 out of 3 mesenchymal markers, one eqiMSC line (FD7) was positive for 1 out of 3 mesenchymal markers, and one eqiMSC line (FD8.6) was negative for all three mesenchymal markers. Two eqBM-MSC lines (D1, D2) were positive for 3 out of 3 mesenchymal markers, and one eqBM-MSC line (D3) was positive for 1 out of 3 mesenchymal markers. Therefore, the MSC-like cells derived from eqiPSCs shared some immunophenotypic features with eqBM-MSCs but also showed certain differences. Notably, both eqiMSCs and eqBM-MSCs showed variability among lines/donors in the expression of certain markers, particularly CD44 and CD105.

### 3.4. Tri-Lineage Differentiation Potential Is Generally Lower in eqiMSCs than in eqBM-MSCs

**Figure 5:**
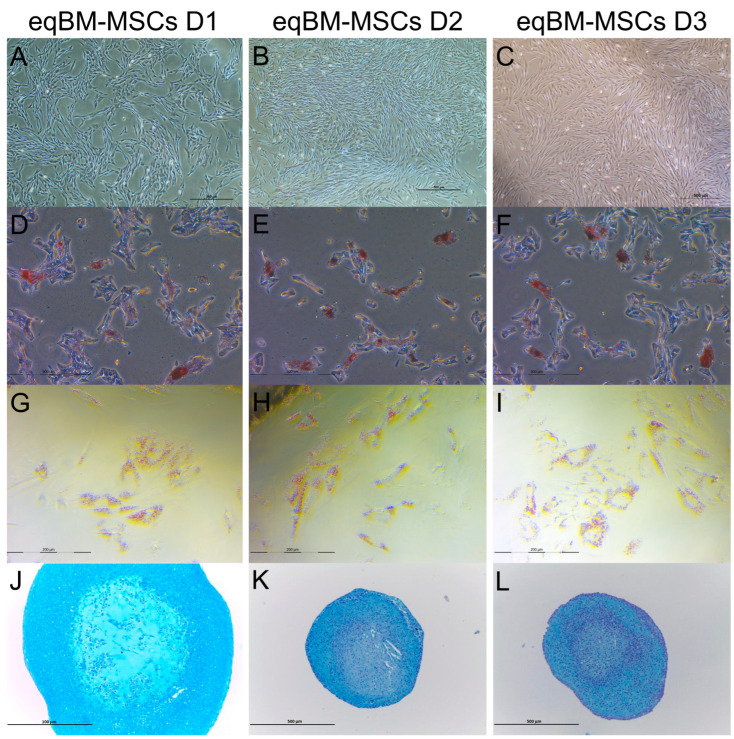
Representative images of the tri-lineage differentiation potential of equine bone marrow-derived mesenchymal stem/stromal cells (eqBM-MSCs) used as a reference in this study. From left column to right column: eqBM-MSCs from the donors D1, D2 and D3. From top row to bottom row: non-differentiated control cells (**A**–**C**), osteogenesis (alizarin red staining) (**D**–**F**), adipogenesis (oil red-O staining) (**G**–**I**), and chondrogenesis (alcian blue staining) (**J**–**L**). Scale bar: (**A**–**F**,**K**,**L**) = 500 µm; (**G**–**I**) = 200 µm; (**J**) = 100 µm.

**Figure 6:**
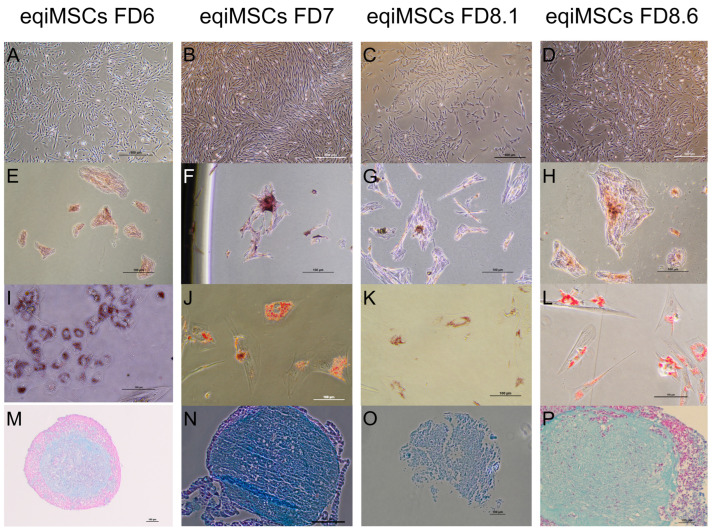
Representative images of the tri-lineage differentiation potential of the equine induced mesenchymal stem/stromal cells-like (eqiMSC) lines obtained in this study. From left column to right column: lines FD6, FD7, DF8.1 and FD8.6. From top row to bottom row: non-differentiated control cells (**A**–**D**), osteogenesis (alizarin red staining) (**E**–**H**), adipogenesis (oil red-O staining) (**I**–**L**), and chondrogenesis (alcian blue staining) (**M**–**P**) differentiations. Scale bar: (**A**–**D**) = 500 µm; (**E**–**P**) = 100 µm.

Equine BM-MSCs (passage 3) and eqiMSCs (passage 6) were subjected to osteogenic, adipogenic and chondrogenic induction in vitro (Figure 5 and Figure 6, respectively). Equine BM-MSCs were used for reference (positive control) (Figure 5), and cells cultured under standard conditions served as non-differentiated negative controls for both eqBM-MSCs and eqiMSCs. These negative control cells did not show any change suggestive of osteogenesis, adipogenesis or chondrogenesis in any case (Figure 5, first row; Figure 5A–C; Figure 6, first row; Figure 6A–D).

For the osteogenic differentiation, the protocol described by our group [43] was used for eqBM-MSCs as customary, while two additional modifications of this protocol were also tested in eqiMSCs with the addition of either BMP7 or CaCl_2_. The latter modification improved the outcome in eqiMSCs. After 9 days under the corresponding osteogenic differentiation conditions, morphological changes, including the acquisition of a polygonal cell shape, were observed in both eqBM-MSCs and eqiMSCs. Overall, changes related to osteogenic differentiation were more noticeable in eqBM-MSCs than in eqiMSCs. Alizarin red staining revealed mineralisation with the formation of calcium deposits in eqBM-MSCs (Figure 5, second row, Figure 5D–F), while there were fewer deposits in eqiMSCs (Figure 6, second row, Figure 6E–H). However, it should be noted that a certain degree of cell detachment occurred during the washing steps prior to staining in osteogenically induced eqiMSCs due to high confluence.

On the other hand, oil red O-stained lipid droplets were detected to a similar extent inside both eqiMSCs and eqBM-MSCs subjected to adipogenic induction over 14 days (Figure 5, third row, Figure 5G–I; Figure 6, third row, Figure 6I–L).

Finally, the sections obtained from the cellular pellets subjected to chondrogenic differentiation for 28 days were stained with alcian blue (Figure 5, fourth row, Figure 5J–L; Figure 6, fourth row, Figure 6M–P). Equine BM-MSCs from the three donors showed similarly marked deposition of proteoglycan-rich extracellular matrix and lacunae formation (Figure 5J–L), but differences in pellet size were noted, with D1 producing larger ones. On the other hand, all four lines of eqiMSCs overall displayed lower chondrogenic potential, with more limited deposition of proteoglycans and lacunae formation, and a less organised extracellular matrix. Notably, inter-line variability in eqiMSCs was particularly noticeable for chondrogenic induction, rather than for osteogenesis and adipogenesis: two eqiMSC lines (FD6, FD8.6) showed moderate chondrogenesis (Figure 6M,P) while one line (FD8.1) was unable to differentiate (Figure 6O).

**Figure 7:**
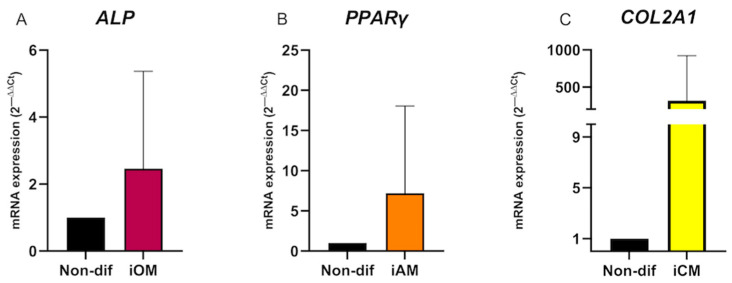
Mean ± S.E.M. of the relative gene expression of lineage-associated genes (**A**) *alkaline phosphatase* (*ALP*, osteogenesis), (**B**) *peroxisome proliferator-activated receptor γ* (*PPARγ*, adipogenesis) and (**C**) *collagen type II* (*COL2A1*, chondrogenesis) in equine induced mesenchymal stem/stromal cells-like differentiated into osteocytes (iOM), adipocytes (iAM) and chondrocytes (iCM) (same lines). Non-differentiated cells from each line (*n* = 4, FD6, FD7, FD8.1, FD8.6) were used as reference samples for normalisation in relative quantification (value 1).

To further characterise the tri-lineage differentiation potential of the eqiMSCs obtained in this study, RT-qPCR was used to assess the expression of lineage-associated genes (Figure 7). An overexpression of *ALP* in iOMs (Figure 7A), *PPARγ* in iAMs (Figure 7B) and *COL2A1* in iCMs (Figure 7C) was observed compared to the corresponding non-differentiated controls. Indeed, non-differentiated controls lacked the expression of these markers in most cases, which prevented the running of comparative statistics.

### 3.5. Gene Expression Complements eqiMSC Characterisation and Shows Changes Along the Differentiation Process

#### 3.5.1. Gene Expression of Cell Surface Markers in eqiNCCs, eqiMSCs and eqBM-MSCs

**Figure 8:**
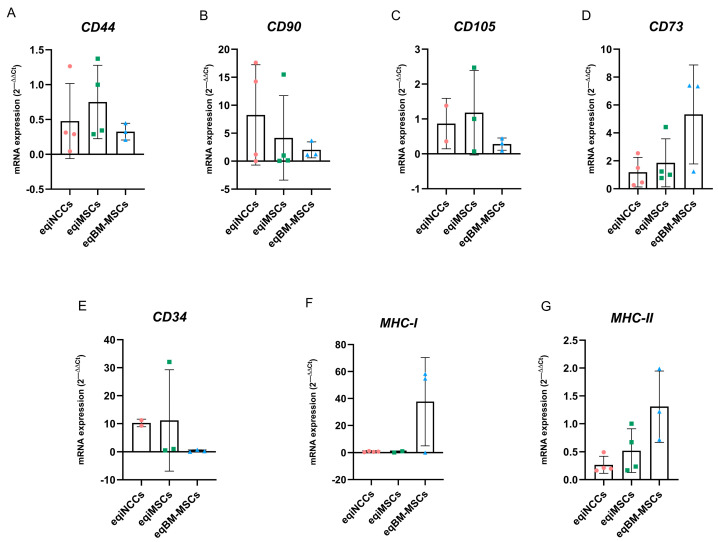
Mean ± S.E.M. of the relative gene expression of the surface markers (**A**) *CD44* (hyaluronate receptor), (**B**) *CD90* (Thy-1), (**C**) *CD105* (Endoglin), (**D**) *CD73* (Ecto-5′-Nucleotidase), (**E**) *CD34* (haematopoietic marker), (**F**) *MHC-I* (major histocompatibility complex class I) and (**G**) *MHC-II* (major histocompatibility complex class II) in eqiNCCs (equine induced neural crest cells; *n* = 4, pink dots), eqiMSCs (equine induced mesenchymal stem/stromal cells-like; *n* = 4, green dots) and eqBM-MSCs (equine bone marrow-derived mesenchymal stem/stromal cells; *n* = 3, blue dots). The eqiMSC line FD8.6 was used as a reference sample for normalization in relative quantification (value 1). No significant differences between cell types were found. *CD45* was analysed, but it was not expressed by any cell type.

To further characterise the novel eqiMSCs, gene expression of cell surface markers was analysed in these cells, along with eqiNCCs and eqBM-MSCs (Figure 8). No significant differences were observed among cell types, which for some markers may be partially explained by the inter-line/donor variability detected in small sample sizes. Nevertheless, some interesting trends were noted, especially for *MHC-I* and *MHC-II*.

In the case of the mesenchymal markers *CD44*, *CD90* and *CD105* gene expression, and contrary to the observations made in terms of surface expression of these molecules, eqBM-MSCs showed lower mean expression than both eqiMSCs and eqiNCCs. However, it should be noted that both eqiMSCs and eqiNCCs showed wider ranges of expression than eqBM-MSCs (Figure 8A–C). Contrary to this pattern, the gene *CD73* (*ecto-5′-nucleotidase*) was expressed at higher mean levels in eqBM-MSCs than in eqiMSCs and eqiNCCs, and more inter-donor variability was observed for eqBM-MSCs as well (Figure 8D).

The haematopoietic marker *CD34* was downregulated in eqiMSCs compared to eqiNCCs, and it was expressed at similar levels between eqBM-MSCs and eqiMSCs, except for one eqiMSC line that expressed higher levels (Figure 8E). The haematopoietic marker *CD45* was also analysed, but it was not expressed by any line from any cell type, and thus it is not represented in Figure 8.

Finally, eqBM-MSCs expressed higher and more variable levels of both *MHC-I* and *MHC-II* compared to the other cell types (Figure 8F,G). Both eqiNCCs and eqiMSCs showed uniformly lower *MHC-I* expression among lines, while *MHC-I* expression varied markedly between eqBM-MSC lines, with one of them displaying close to null expression and two expressing higher relative values. Regarding *MHC-II*, its lowest expression was detected in eqiNCCs, followed by eqiMSCs, whereas eqBM-MSCs comparatively showed the highest levels.

#### 3.5.2. Gene Expression of Pluripotency Markers in eqiPSCs, eqiNCCs, eqiMSCs, iAM, iCM and iOM

**Figure 9:**
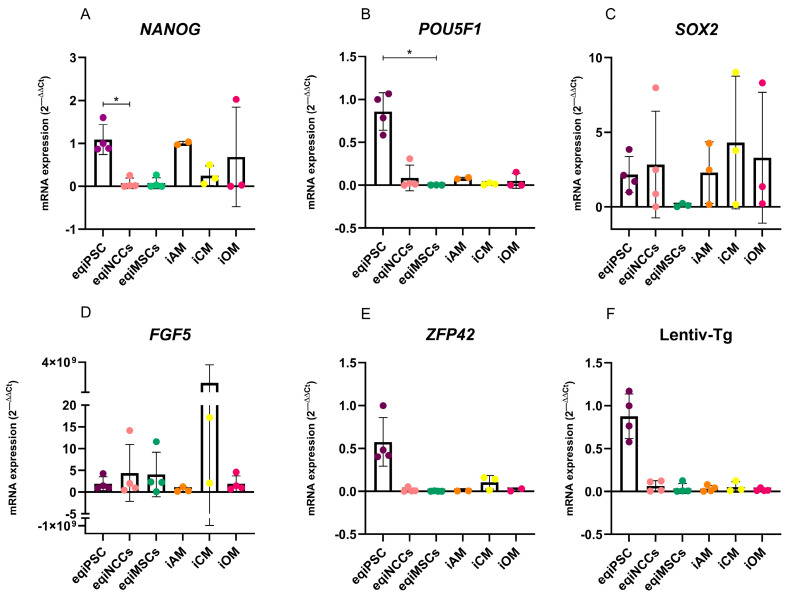
Mean ± S.E.M. of the relative gene expression of pluripotency-associated genes (**A**) *NANOG* (homeobox protein), (**B**) *POU5F1* (POU class 5 homeobox 1), (**C**) *SOX2* (SRY-Box Transcription Factor 2), (**D**) *FGF5* (fibroblast growth factor 5), (**E**) *ZFP42* (Zinc Finger Protein 42) and (**F**) of the lentiviral transgene (Lentiv-Tg) in paired lines of eqiPSCs (equine induced pluripotent stem cells; *n* = 4, purple dots), eqiNCCs (equine induced neural crest cells; *n* = 4, light pink dots), eqiMSCs (equine induced mesenchymal stem/stromal cell-like; *n* = 4, green dots), iAM (adipocytes differentiated from eqiMSC; *n* = 4, orange dots), iCM (chondrocytes differentiated from eqiMSCs; *n* = 4, yellow dots) and iOM (osteocytes differentiated from eqiMSCs; *n* = 4, dark pink dots). The eqiPSC line FD8.6 was used as a reference sample for normalisation in relative quantification (value 1). Significant differences between experimental conditions are represented by a squared line with an asterisk (* = *p* < 0.05).

To better understand the changes experienced by the cells along the entire differentiation process, the relative expression of pluripotency-associated genes was analysed in the following cell types: the original eqiPSCs, the intermediate eqiNCCs, the resulting eqiMSCs, and in the iAM, iCM and iOM derived from the tri-lineage differentiation of eqiMSC (Figure 9). Statistically significant downregulation of the core pluripotency markers *NANOG* and *POU5F1* was detected, respectively, in eqiNCCs compared to eqiPSCs (*p* = 0.04) (Figure 9A) and in eqiMSCs compared to eqiPSCs (*p* = 0.03) (Figure 9B).

In the other cell types and for the other genes, no statistically significant differences were detected, but relevant changes were observed. The expression of *NANOG*, *POU5F1* and *ZFP42* (*zinc finger protein 42*, naïve pluripotency marker) was greatly downregulated in both eqiNCCs and eqiMSCs compared to eqiPSCs (Figure 9A,B,E). On the other hand, *FGF5* (*fibroblast growth factor 5*, primed pluripotency marker) was upregulated in eqiNCCs and eqiMSCs compared to eqiPSCs (Figure 9D). Finally, *SOX2* (*SRY-box transcription factor 2*) expression widely varied among all the cell types, showing an upregulation in eqiNCCs compared to eqiPSCs, but a downregulation in eqiMSCs compared to eqiPSCs (Figure 9C).

The expression of *POU5F1* and *ZFP42* remained downregulated in the differentiated adipocytes (iAM), chondrocytes (iCM) and osteocytes (iOM) derived from eqiMSCs (Figure 9B,E). On the other hand, the expression of *NANOG* in iAM and in one line of iOM presented an upregulation compared to both eqiMSCs and eqiNCCs, but the mean expression level remained below that of eqiPSCs (Figure 9A). An upregulation of *SOX2* was detected in some lines of iAM, iCM and iOM compared to the preceding eqiMSCs (Figure 9C). In terms of *FGF5* expression, iAM and iOM showed downregulation compared to the precedent cell stages (eqiPSCs, eqiNCCs, eqiMSCs), but iCM showed a marked upregulation of this gene in two lines (Figure 9D).

Finally, we observed a clear and sustained downregulation in the expression of the transgene used to reprogram eqiPSCs [27] across the differentiated cell types (eqiNCCs, eqiMSCs, iAM, iCM, iOM) compared to eqiPSCs (Figure 9F).

## 4. Discussion

This study demonstrates that equine MSC-like cells (termed eqiMSCs) can be obtained from eqiPSCs using a novel differentiation protocol adapted from human literature. In contrast to previously reported methods in the equine species that are based on spontaneous derivation [15,18], our approach consisted of directing the differentiation via the neural crest pathway. Using this protocol, the derivation of eqiMSCs could be reproduced in all four eqiPSC lines tested. The majority of the resulting cell lines met most of the standard MSC characterisation criteria, but inter-line variability was noted, and some eqiMSC lines did not fulfil all the criteria, displaying differences compared to the eqBM-MSCs used for reference. Importantly, such variability—both among iMSC lines and in comparison to primary MSCs—has also been documented in human studies without compromising functional potency [34]. Indeed, Palamá et al. [34] found that human iMSCs secrete higher levels of anti-inflammatory extracellular vesicles despite showing greater variability and less consistent tri-lineage differentiation than human BM-MSCs. Even though the behaviour of these cells cannot be directly extrapolated across species, the variability observed in this study could be interpreted within the context of the biology of iPSC-derived MSC-like cells [33,50,51,52,53].

The therapeutic effects of MSCs present in diverse tissues make them highly attractive for treating different conditions in both human and veterinary patients [3]. However, since these cells pose important limitations for the scalable and sustainable use of cell therapy, MSC-like cells derived from iPSCs (iMSCs) have been suggested as an attractive alternative source of therapeutic cells [14]. To this end, iMSCs are being intensively investigated in human medicine [16], emphasising the importance of also developing this approach in the veterinary field, where advancement has been considerably limited. Although there is a growing interest in developing iMSCs in other animal species such as canine, feline or even Tasmanian devil [33,50,51,52,53], there are only two works published in the equine species [15,18].

Methods to obtain iMSCs can be generally classified as either spontaneous differentiation or directed differentiation through a specific pathway [19]. Human iMSCs have been derived from iPSCs using both spontaneous differentiation [54] and directed differentiation through different signalling pathways [9,22,55]. However, the only two published works reporting the obtainment of equine iMSCs followed spontaneous differentiation strategies [15,18], but used different protocols. Given the relative simplicity of these protocols, we started testing both of them in our eqiPSCs. However, none of these yielded consistent results under our conditions (Supplementary Material S1).

It is difficult to elucidate the reasons for the failed differentiation of the eqiPSCs used in this study when following the protocols previously reported in the equine species. One potential explanation considers the different characteristics of eqiPSCs among studies, since different equine cell types might yield iPSCs of distinct characteristics, which may also differ in their predisposition for subsequent differentiation [10,56]. Studies in the feline species have also described poor outcomes with spontaneous protocols to obtain iMSCs [33]. These observations highlight the lack of standardised procedures and the need for further research to understand both iPSC and iMSC biology in veterinary species.

In order to address this issue, we decided to explore directed differentiation approaches. First, we attempted a protocol previously described for directing the differentiation of human iPSCs via the LPM pathway [22]. The rationale for this choice was that BM-MSCs mostly originate from the mesoderm, and appendicular bones derive specifically from the LPM [20,21]. Thus, we tested the protocol described by Wei et al. (2022) [22] with eqiPSCs, but these were unable to navigate the differentiation process and collapsed very soon during the first stages of induction (Supplementary Material S1). Subsequently, we decided to explore another directed approach by using the neural crest pathway. The neural crest is a transient population of multipotent stem cells with the ability to give rise to a wide range of cell types and tissues [57]. Actually, even though most MSC populations in the organism derive from the mesoderm, there is a population that derives from the ectoderm via the neural crest to originate the skeletal structures of the head. Based on this approach, we adapted a protocol from human literature [9,24,25,26] to obtain eqiMSCs.

The main adjustment required in the protocol was due to the impossibility of starting the induction of differentiation of eqiPSCs in feeder-free conditions. While efforts were made to grow our eqiPSCs on basement membranes (gelatine, matrigel, vitronectin), these cells eventually collapsed in feeder-free conditions, making it impossible to induce their differentiation in this culture system. In many laboratories, iPSCs are maintained on animal-derived feeder cells such as iMEFs [58], particularly in the case of companion animal species [51,59,60]. Feeder layers secrete a variety of growth factors involved in maintaining the pluripotent and undifferentiated state of pluripotent stem cells (PSCs), as well as in promoting their growth and attachment [61]. Indeed, most reports on eqiPSCs describe the use of feeder cell layers (e.g., iMEFs). Given the still limited understanding of equine iPSC biology, standardised systems for feeder-free culture have not yet been thoroughly established [10], even though promising advancements have been made recently [62]. In this situation, we opted to culture eqiPSCs on iMEF layers during the first stage of the differentiation protocol in order to ensure the initial adherence and vitality of eqiPSCs. While the signalling provided by iMEFs can contribute to maintaining pluripotency, there is previous evidence that PSCs can differentiate in the presence of feeder cells [61].

The differentiation protocol reported here encompassed two stages: obtainment of an intermediate population of eqiNCCs and inducing them into MSC-like cells (eqiMSCs). All four eqiPSC lines tested in this study yielded intermediate eqiNCC populations characterised by the expression of the neural crest markers *RHOB* and *NES* [47,48]. It should be noted that NCC identity should be interpreted in the context of a limited set of transcriptional markers, warranting further investigation of their profile and species-specific features. In addition, the same MSC markers assessed in eqiMSCs were also studied in eqiNCCs. While each line presented individual variations in the evolution of these markers, an average trend could be observed—both by flow cytometry and gene expression: cells transitioning from eqiNCCs to eqiMSCs maintained similar levels of CD44, decreased CD90 levels and increased CD105 levels. Regarding MHC-I and MHC-II expression in eqiNCCs, it was similar to that in eqiMSCs and lower than in eqBM-MSCs, both at the surface and at the gene expression levels. The lower expression of MHC-I in both eqiNCCs and eqiMSCs might reflect that these cells present a lower differentiation state compared to adult eqBM-MSCs, as it has been described that stem cells in less differentiated stages display reduced MHC-I expression [63].

In the second stage of the directed differentiation process, the four lines of eqiNCCs could be induced into four lines of MSC-like cells (eqiMSCs), which were characterised based on morphology, marker expressions and tri-lineage differentiation. The use of four independent eqiMSC lines is consistent with recent reports in humans [9,34] and expands upon earlier studies in the equine field. Previous equine studies focused on characterising single eqiMSC lines and used a more limited set of markers and lineage specifications [15,18]. Building on these foundational studies, the present work enables the assessment of inter-line variability and incorporates a broader panel of characterisation criteria, providing a more comprehensive understanding of eqiMSC properties.

The eqiMSCs obtained in the current study presented a morphology very similar to that of primary eqBM-MSCs, but differences were observed in their immunophenotype and in their tri-lineage differentiation potential. Equine iMSCs overall showed lower percentages of positive cells than eqBM-MSCs for the mesenchymal surface markers, but eqiMSCs were still positive for CD44 and CD90, except for one line, FD8.6, which was negative for all the mesenchymal markers analysed. However, the tri-lineage differentiation potential of the FD8.6 eqiMSC line was higher than that of the eqiMSC line FD8.1, which expressed the highest levels of CD44 and CD105 among all eqiMSC lines but presented the most limited chondrogenic potential. These observations support the notion that standard characterisation criteria may not fully reflect the functional potency of eqiMSC lines, as previously suggested in human studies [34]. This warrants further research into the properties of these novel eqiMSCs to generate species-specific knowledge, as the interpretation of results in the frame of human evidence should be done carefully.

A potential explanation for the observed inter-line variability in eqiMSCs is that independent eqiPSC lines might undergo mesenchymal commitment with distinct temporal kinetics. This would imply that differentiation may be intrinsically asynchronous, rather than reflecting a lack of robustness in the protocol tested in this study. The eqiMSC lines obtained in this study derive from four different eqiPSC lines, generated from a single donor in the same reprogramming experiment but monoclonally expanded, thus presenting comparable characterisation while showing subtle variations. Indeed, genetic and epigenetic variations among different iPSC lines can originate during reprogramming [64] and may have an effect on their differentiation properties [65]. It is worth noting that the expression of the transgene was slightly lower in the eqiPSC line FD8.1, while the expression of the naïve pluripotency marker *ZFP42* (Rex1) was slightly higher in the eqiPSC line FD8.6 [27]. Such expression patterns might have, respectively, favoured or prevented the differentiation of eqiPSCs into MSC-like cells, potentially leading to more marked differences between eqiMSC lines FD8.1 and FD8.6. While it is not possible to establish this correlation with the available data, these observations warrant further transcriptomic investigation to link features of eqiPSCs with the characteristics of their progeny and to explore it as a source of inter-line variability.

Related to this, another potential source of inter-line variability may be associated with the cells’ passage number. In line with this, but in a different species, recent human studies have reported significant variability not only among distinct iMSC lines but also across different passages of the same line [34]. The analysis of eqiMSCs at passage 6 in this study might be capturing a transitional stage of differentiation, with some lines being assessed at different maturation states. The rationale for selecting passage 6 for characterisation aligns with the criteria used in studies conducted in other species, which characterised iMSCs once these showed a morphology and growth behaviour similar to that of primary MSCs [33]. This provides a benchmark for comparison despite the inherent difficulty in defining a single point, but implies that certain eqiMSC lines might not have yet reached full maturity at passage 6. This constitutes a limitation of this study, and future work should look into serial characterisation across passages. This would help to resolve the contribution of the stabilisation process to the inter-line variability observed in equine iMSCs.

In addition to these considerations, it should also be noted that the expression of MSC surface markers can be heterogeneous among cell sources and across different species [66]. Variability can also be attributed to the individual, as in the case of CD90, which in equine BM-MSCs can range between 30% and 95% depending on the donor [67]. The mesenchymal marker CD105 was not expressed by eqiMSCs, except for the line FD8.1, but other reports in equine primary MSCs, specifically in cord blood-derived MSCs, have described the lack of expression of this marker as well [15,26]. Moreover, from the two previous studies on eqiMSCs, one of them showed that only 1.14% of eqiMSCs were CD105+ [15], whereas the other study did not include the analysis of this marker [18]. Additionally, CD105 has been reported as a marker of iMSC maturation in human studies [68]. While this observation cannot be directly extrapolated to equine, it would match with the average increase of this marker in eqiMSCs compared to eqiNCCs. Additionally, it was also noted that the line FD8.1 displayed a more mature phenotype and presented the highest expression of CD105.

In order to gather further information about the characteristics of eqiMSCs, the same markers were analysed at the gene expression level. While the expression of the genes coding for the mesenchymal markers *CD44*, *CD90*, *CD105* and *CD73* also showed inter-line variability, their average levels in eqiMSCs were similar to, or even higher than, in eqBM-MSCs. While we do not have a clear explanation for this observation, it is not uncommon to find discrepant results between gene and protein expression [46], which might be due to posttranscriptional mechanisms or external factors affecting surface antigen expression. Regardless of the underlying reason, the expression of genes coding for these markers in eqiMSCs supports their induction into mesenchymal cells.

In the case of haematopoietic markers, eqiMSCs met the criteria of being negative for CD11α/18 and CD45 by flow cytometry. The marker CD34 was assessed in terms of gene expression due to the lack of suitable antibodies that worked under our conditions. We observed higher mean levels of *CD34* in eqiMSCs compared to eqBM-MSCs, but this was due to expression of this gene in a single eqiMSC line (FD6). The variability in the gene expression of MSC characterisation markers has been reported in equine MSCs from different tissues [37]. For instance, Ranera et al. (2011) [46] showed positive gene expression of *CD34* in equine adipose tissue-derived MSCs, while it was not expressed by eqBM-MSCs.

Finally, and as aforementioned, the expression of the antigen-presenting molecules MHC-I and MHC-II, as assessed both at the surface and at the gene expression levels, was lower in eqiMSCs compared to eqBM-MSCs. This might suggest that eqiMSCs could potentially be less immunogenic than eqBM-MSCs. The effect of MHC-I and MHC-II expression on the immune recognition of equine MSCs is well documented, both in vitro [67] and in vivo [29], with higher expression leading to higher activation of T and B cells [28,69]. While current evidence is still preliminary to draw definitive conclusions, it opens interesting avenues to explore the interaction of eqiMSCs with the immune system. This is particularly important considering that the use of these cells would mainly occur in an allogeneic context.

Overall, there were some dissimilarities between the eqiMSCs obtained in this study and the eqBM-MSCs used as a reference. Beyond the aspects discussed above regarding the maturation stage of eqiMSCs, it should also be noted that there is an intrinsic heterogeneity of MSCs across tissue sources [37], as they originate from different germ layers. The mesoderm is the main contributor to these populations, but there are also MSC populations in the craniofacial region that arise from the ectoderm via the neural crest. Because of their different origins, these cells show transcriptional and functional differences [70]. In particular, the neural crest is a source of MSCs specialised in supporting the hematopoietic stem cell niche, with a more limited contribution to osteochondrogenesis [20]. Actually, canine BM-MSCs derived from jaw bone showed lower chondrogenic potential than BM-MSCs isolated from the ilium [66]. Similarly, the pathway through which the eqiMSCs in this study were derived may be influencing their characteristics, resulting in cells that are not equivalent to mesoderm-derived BM-MSCs but instead present a distinct profile.

The eqiMSCs obtained in our study exhibited adipogenesis similar to eqBM-MSCs, but their osteogenic potential was more limited, even though fine-tuning was pursued through the addition of CaCl_2_ to the osteogenic media. Osteogenesis of eqBM-MSCs was induced using the protocol customarily used by our group for these cells [43], in order to serve as an internal control for standard osteogenesis of equine MSCs. This variation in media composition prevented straight comparison of the osteogenic potential between MSC types, constituting a limitation of this study. Another technical limitation for the interpretation of eqiMSC osteogenesis outcome was the partial detachment of these cells during the washing steps prior to staining. This was attributed to the high confluence of cells by the end of the osteogenesis induction, so cell density optimisation for this assay should be pursued in future works. In the light of these considerations, and despite moderate staining with alizarin red, eqiMSCs showed morphological changes, produced small calcium deposits and upregulated *ALP* gene expression, all of them being indicative of osteogenic induction. Variable osteogenic potential has also been observed in equine primary MSCs derived from different tissues. For example, equine adipose tissue-derived MSCs present lower capacity than eqBM-MSCs to undergo osteogenic differentiation [45].

The same rationale may underlie the variable chondrogenic potential in MSCs [71], which in our study was lower in eqiMSCs compared to eqBM-MSCs. Variability was also observed among lines, with one of them (line FD8.1) completely failing chondrogenic induction in spite of showing an immunophenotype suggestive of a more mature state. On the contrary, the eqiMSC line FD8.6, despite not expressing any of the mesenchymal surface markers (CD44, CD90, CD105), showed tri-lineage differentiation ability, including higher chondrogenic potential than other lines. Interestingly, progenitor MSCs may not express CD44 in vivo and instead acquire it during in vitro expansion [72]. Similarly, the expression of CD90 has been reported to be lower at early passages, and increase over time [73]. Moreover, the expression of mesenchymal markers is not always associated with the differentiation ability of MSCs. For instance, both CD105- and CD105+ populations of human AT-MSC and umbilical cord-MSC have shown comparable tri-lineage differentiation ability [74]. Moreover, it has been suggested that the neural crest origin of primary MSCs, such as those associated with dental structures, might present limited ability to form hyaline cartilage [23]. The quantification of extracellular matrix components in the eqiMSC chondrogenic pellets might have provided further cues on this potential, and the lack of such assessments constitutes another limitation of the current study.

Finally, to better understand the changes in the pluripotent profile of the cells along the differentiation process, the expression of pluripotent-related genes was assessed in all the cells generated: eqiPSCs, eqiNCCs, eqiMSCs, and their derived adipocytes, osteocytes and chondrocytes. In general, we observed a downregulation of these genes along the subsequent differentiation stages, particularly from the initial eqiPSCs, especially for the core markers *NANOG* and *POU5F1* (Oct4), and the naïve marker *ZFP42* (Rex1). On the contrary, the primed marker *FGF5* tended to increase in eqiNCCs and eqiMSCs compared to eqiPSCs. It should be noted that the line FD6 was the main contributor to the average increase of *SOX2* in eqiNCCs compared to eqiPSCs, and in the tri-lineage differentiated cells over eqiMSCs. Notably, the chondrogenic lineage expressed the highest *SOX2* levels among the differentiated cell types. *SOX2* is a well-established regulator of pluripotency and stem cell maintenance, and sustained expression of this factor has been associated with reduced differentiation capacity in mesenchymal lineages [75]. In this context, the elevated *SOX2* levels observed in our chondrogenic cultures could suggest persistence of a less differentiated or progenitor-like state, potentially contributing to the limited chondrogenic differentiation observed. However, this interpretation remains speculative and leaves room for other interpretations based on context-dependent roles of this regulatory transcription factor, warranting further studies to clarify the significance of *SOX2* in eqiMSC chondrogenesis. We also confirmed that the expression of the transgene used to reprogram eqiPSCs [27] was greatly and consistently downregulated in the eqiNCCs and eqiMSCs. The transgene expression was maintained at the same low levels in the adipocytes, chondrocytes and osteocytes derived from eqiMSCs.

Altogether, these observations reinforce the notion that directing the cells through the neural crest pathway can force eqiPSCs to leave their pluripotent state and enter the differentiation process. However, the downregulation of core pluripotent genes (*POU5F1*, *NANOG*, *SOX2*) and of the lentiviral transgene in eqiMSCS would not be enough to confirm that all lines have reached the same differentiation stage. While the expression of these genes was markedly reduced in all the eqiMSC lines, some intermediate eqiNCC lines showed higher levels of *POU5F1*, *SOX2* and *FGF5* than others. This might reflect different states of differentiation that are later translated into varying maturation of eqiMSCs. While these observations are still too preliminary to draw any definitive conclusions, they highlight the need for further transcriptomic research to shed light on the changes experienced by cells navigating this differentiation process.

## 5. Conclusions

This study presents for the first time that MSC-like cells (iMSCs) can be derived from equine iPSCs by directing their differentiation through the neural crest pathway. This method of directed differentiation represents a feasible option for eqiMSC generation, thus constituting an important step toward more sustainable sources of therapeutic cells in veterinary medicine. The majority of the eqiMSC lines obtained met most of the standard criteria defined for tissue-derived MSCs, but showed some differences compared to primary equine MSCs and displayed certain variability across lines: the former finding can reflect their different developmental origin, while the latter observation might suggest differences in differentiation kinetics despite using the same protocol, thus also pointing out intrinsic characteristics of the originating iPSC lines. While the use of equine iPSCs to obtain therapeutic cells opens promising avenues, further research is warranted to gain insight into the transcriptomic changes that cells undergo during differentiation. The present work provides a comprehensive characterisation of the mesodermal profile of these novel cells as a necessary first step. Further research is required to explore the functional attributes of eqiMSCs, particularly in terms of advantages for expansion potential, therapeutic mechanisms and immunological profile.

## Figures and Tables

**Table 1:** Antibodies used for flow cytometry characterisation of equine cells.

Antibody	Manufacturer and Reference	Host and Target Species	Fluorophore	Clone and Isotype	References
CD44	BioRad MCA1082PE (Barcelona, Spain)	Mouse anti-horse	RPE	CVS18 isotype IgG1	[38,39,40]
CD90	BD Pharmigen 555596 (Fisher Scientific, Madrid Spain)	Mouse anti-human	RPE	5E10 isotype IgG1	Equine cross-reactivity tested by our group
CD105	BioRad MCA1557A647	Mouse anti-human	Alexa Fluor 647	SN6 isotype IgG1	[40,41]
CD45	BioRad MCA87A700	Mouse anti-human	Alexa Fluor 700	F10-89-4 isotype IgG2a	Equine cross-reactivity tested by our group
CD11α/CV18	BioRad MCA1081PE	Mouse anti-horse	RPE	CVS9 isotype IgG1	[39]
MHC-I	BioRad MCA1086PE	Mouse anti-horse	RPE	CVS22 isotype IgG2a	[42]
MHC-II	BioRad MCA1085F	Mouse anti-horse	FITC	CVS20 isotype IgG1	[38,39]

**Table 2:** GenBank accession number of the sequences used for primer design.

Gene	Accession Number	Primer Sequence (5′–3′)	Amplicon Size (bp)
House-keeping			
*GAPDH*	NM_001163856	F: GGCAAGTTCCATGGCACAGT R: CACAACATATTCAGCACCAGCAT	128
*B2M*	NM_001082502.2	F: TCGTCCTGCTCGGGCTACT R: ATTCTCTGCTGGGTGACGTGA	102
Neural crest cell markers			
*RHOB*	XM_005600187.4	F: GTAAGGACGAGTTCCCCGAG R: GGGGATGTTCTCCAGCGAAT	212
*NES*	XM_023640985.2	F: CAAATCGCCCAGGTCCTG R: GCCTCTAGGAGGGTCCTGTATGT	95
Characterisation positive markers (mesenchymal markers)			
*CD44*	NM 001085435	F: CCCACGGATCTGAAACAAGTG R: TTCTGGAATTTGAGGTCTCCGTAT	95
*CD90*	EU881920	F: TGCGAACTCCGCCTCTCT R: GCTTATGCCCTCGCACTTG	93
*CD105*	XM_001500078	F: GACGGAAAATGTGGTCAGTAATGA R: GCGAGAGGCTCTCCGTGTT	100
*CD73*	XM 001500115	F: GGGATTGTTGGATACACTTCAAAAG R: GCTGCAACGCAGTGATTTCA	90
Characterisation negative markers (haematopoietic markers)			
*CD34*	XM_001491596	F: CACTAAACCCTCTACATCATTTTCTCCTA R: GGCAGATACCTTGAGTCAATTTCA	150
*CD45*	AY_114350	F: TGATTCCCAGAAATGACCATGTA R: ACATTTTGGGCTTGTCCTGTAAC	100
Antigen presenting-related molecules			
*MHC-I*	AB525081	F: CGTGAGCATCATTGTTGGC R: TCCCTCTTTTTTCACCTGAGG	92
*MHC-II*	NM_001142816	F: AGCGGCGAGTTGAACCTACAGT R: CGGATCAGACCTGTGGAGATGA	172
Osteogenic marker			
*ALP*	XM_001504312	F: GATGGCCTGAACCTCATCGA R: AGTTCGGTCCGGTTCCAGAT	92
Adipogenic marker			
*PPARγ*	XM_001492411	F: TGCAAGGGTTTCTTCCGGA R: GCAAGGCATTTCTGAAACCG	104
Chondrogenic marker			
*COL2A1*	XM_005611082.1	F: TTAGACGCCATGAAGGTTTTCTG R: CTCTTGCTGCTCCACCAGTTCT	101
Pluripotency markers			
*NANOG*	XM_023643093.1	F: CTCGATTTGGGCAGTGGCTA R: CGAGCCCTCTAGAATCCGTC	117
*POU5F1 (Oct4)*	XM_023624232.1	F: AGAAGGACGTGGTACGAGTG R: GTGCCAGGGGAAAGGATACC	138
*SOX2*	XM_023623361.1	F: CCATTAACGGCACACTGCCC R: AGAATTTCTCCCCCACCTCCAG	72
*FGF5*	XM_014738875.220	F: GACCCGTTGCCACTGATAGG R: TCGTGGGAGCCATTGACTTT	250
*ZFP42 (Rex1)*	XM_001489519.4	F: TGGAGGAATATCCAGCGTTGA R: GCTTTCCCACATTCTGCACATA	213
Transgene			
Lentiv-Tg	Sequence from supplier	F: CCACCTCGCCTTACACATGA R: TGCTGGTTTTCCACTACCCG	141

Primers (F: forward and R: reverse) and length of the amplicon in base pair (bp). Genes were grouped in accordance with the functions and implications of encoded molecules. *GAPDH*, *glyceraldehyde 3-phosphate dehydrogenase; B2M, beta-2 microglobulin; RHOB, Ras homolog family member B; NES, Nestin; CD44, hyaluronate receptor; CD90, Thy-1; CD105, endoglin; CD73, ecto-5′-nucleotidase; CD34, hematopoietic marker; CD45, hematopoietic marker; MHC-I and MCH-II, major histocompatibility complex I and II; ALP, alkaline phosphatase; PPARγ, peroxisome proliferator-activated receptor ϒ; COL2A1, collagen type II; NANOG, homeobox protein; POU5F1, POU class 5 homeobox 1* (coding Oct4)*; SOX2, SRY-box transcription factor 2; FGF5, fibroblast growth factor 5; ZFP42, zinc finger protein 42* (coding Rex1).

**Table 3:** Surface marker expression in eqiNCCs, eqiMSCs, and eqBM-MSCs assessed by flow cytometry.

Cell Type	Cell Line	CD44	CD90	CD105	CD45	CD11α/CD18	MHC-I	MHC-II
eqiNCCs	FD6	64.96	99.87	0.87	0.35	0.05	49.66	0.0
	FD7	17.15	93.21	0.62	1.23	0.35	14.36	0.15
	FD8.1	96.89	5.41	25.37	0.38	0.76	60.36	1.25
	FD8.6	3.76	80.14	0.02	0.03	0.02	2.07	0.13
eqiMSCs	FD6	31.66	96.83	0.93	11.28	0.21	23.61	0.62
	FD7	40.85	10.35	0.7	0.07	0.38	2.49	0.1
	FD8.1	68.99	19.11	53.92	2.23	1.38	8.45	0.61
	FD8.6	0.02	0.01	0.51	0.63	6.08	0.0	0.73
eqBM-MSCs	D1	89.575	98.445	87.615	0.1	0.235	15.32	1.39
	D2	92.565	97.325	88.695	0.005	0.06	87.4	0.625
	D3	17.135	73.32	11.73	0.005	0.01	41.515	0.085

## Data Availability

The raw experimental data that support the findings of this study are available in Zenodo with the identifiers https://doi.org/10.5281/zenodo.20035832 and https://doi.org/10.5281/zenodo.20040545.

## Supplementary Materials

The following supporting information can be downloaded at https://www.mdpi.com/article/10.3390/ani16111618/s1, Supplementary Material S1: Approaches to obtain equine MSC-like cells from equine iPSCs via spontaneous differentiation. Supplementary Material S2: Representative gating plots for the analysis of CD90 and CD105.

## Abbreviations

AF: attachment factor; ALP: alkaline phosphatase (gene); B2M: beta-2 microglobulin; bFGF: basic fibroblast growth factor; BMP7: bone morphogenetic protein 7; BM-MSCs: bone marrow derived MSCs; CD44: hyaluronate receptor; CD90: Thy-1; CD105: endoglin; CD73: ecto-5′-nucleotidase; CD11α/CD18: hematopoietic marker; CD34: hematopoietic marker; CD45: hematopoietic marker; CDLC: chemically defined lipid concentrate; cDNA: complementary DNA; COL2A1: collagen type II; DMEM: Dulbecco’s modified Eagle’s medium; DMSO: dimethyl sulfoxide; EGF: epidermal growth factor; eqBM-MSCs: equine BM-MSCs; eqiMSCs: equine iMSCs; eqiNCCs: equine iNCCs; eqiPSCs: equine iPSCs; FBS: foetal bovine serum; FGF5: fibroblast growth factor 5 (gene); GAPDH: glyceraldehyde 3-phosphate dehydrogenase (gene); iAM: adipocytes differentiated from eqiMSCs; IBMX: 3-isobutyl-1-methylxanthine; iCM: chondrocytes differentiated from eqiMSCs; IMDM: Iscove’s modified Dulbecco’s medium; iMEFs: irradiated mouse embryonic fibroblasts; iMSCs: induced MSCs; iNCCs: induced NCCs; iOM: osteocytes differentiated from eqiMSCs; iPSCs: induced pluripotent stem cells; ITS: insulin transferrin selenium; IV: intravenous; KOSR: knockout serum replacement; LIF: leukemia inhibitory factor; LPM: lateral plate mesoderm; MHC-I: major histocompatibility complex I; MHC-II: major histocompatibility complex II; MSCs: mesenchymal stem/stromal cells; NANOG: homeobox protein (gene); NCCs: neural crest cells; NEAA: non-essential amino acids; PBS: phosphate-buffered saline; PFA: paraformaldehyde; POU5F1: POU class 5 homeobox 1 (gene); PPARϒ: peroxisome proliferator-activated receptor ϒ (gene); PSC: pluripotent stem/stromal cell; RT-qPCR: real-time quantitative polymerase chain reaction; SOX2: SRY-box transcription factor 2 (gene); TGFβ-3: transforming growth factor-beta 3; ZFP42: zinc finger protein 42 (gene).
