# Supplementary material for: Derivation of Equine Mesenchymal Stem/Stromal Cells from Induced Pluripotent Stem Cells via the Neural Crest Pathway and Characterisation by Immunophenotype and Tri-Lineage Differentiation
Source: Animals (Basel). 2026 May 26;16(11):1618. doi: 10.3390/ani16111618 (PMC13255804; doi:10.3390/ani16111618)
Supplement: Supplementary file 1 [file animals-16-01618-s001.zip › Supplementary Material S1.pdf]

Supplementary Material S1: Approaches to obtain equine MSC-like cells from equine iPSCs via spontaneous differentiation

1. Introduction

Prior to implementing the protocol described in the main manuscript to obtain MSC-like cells from eqiPSCs (equine induced pluripotent stem cells), we first tested the only two published protocols in the equine species. Both protocols, , termed as Protocol 1 [1] and Protocol 2 [2], were based on the spontaneous differentiation of eqiPSCs. In addition, a protocol based on directing the differentiation of eqiPSCs through the lateral plate mesoderm (LPM) was also tested, using a method previously described for human cells [3]. In our conditions, none of these protocols yielded satisfactory results, but the approaches used and the observations made along the process are here presented to guide future attempts.

2. Methodology

One line of eqiPSCs (named FD6) was used as a pilot to test these three protocols. The eqiPSC line was generated in a previous study [4] and used in passage 14, using the culture conditions described in the main manuscript.

2.1. Spontaneous differentiation protocols (Protocol 1 and Protocol 2)

For the first protocol (Protocol 1, described by Chung et al., (2019) [1]), eqiPSCs were detached by enzymatic digestion as described in the main manuscript and seeded into 6-well plates, either onto vitronectin (Gibco™, Thermo Fisher) or onto irradiated mouse embryonic fibroblasts (iMEFs). Vitronectin coating was initially tested, as reported in the original protocol [1], but the poor adaptation of the eqiPSCs used in this study to feeder-free conditions led us to also attempt the differentiation using iMEFs to initially support eqiPSCs attachment and vitality. In both cases, the culture media was directly switched to standard MSC culture medium described in the main manuscript, and supplemented with 10 µL/mL of Revitacell™ (100X) (Gibco™, Thermo Fisher) for the first 24 hours. The three first cell passages were performed by enzymatic digestion using StemPro Accutase (Gibco™, Thermo Fisher) every 5-7 days. Subsequently, cells were passaged by enzymatic digestion with Trypsin-EDTA (0.25 %) (Gibco™, Thermo Fisher) and seeded using a 1:2 ratio into 175 cm^2 tissue culture treated flasks with no coating, and maintained in the same growth medium.

In the second protocol (Protocol 2, described by Lepage et al., (2016) [2]), eqiPSCs were detached by enzymatic digestion and seeded at 1:1 ratio onto 6-well plates coated with attachment factor (AF) but with no iMEFs. The cells were grown in medium consisting of high glucose Dulbecco's Modified Eagle Medium (DMEM; Gibco™, Thermo Fisher), 10 % foetal bovine serum (FBS), 0.1 mM non-essential amino acids (NEAA), 100 U/mL penicillin, 100 µg/mL streptomycin, 10 µM of SB431542 (TGF-β receptor kinase inhibitor, Quimigen, MedChemExpress) and 5 ng/mL of basic fibroblast growth factor (bFGF 154 a.a; Quimigen, MedChemExpress). Cells were maintained in these conditions for 10 days, with one enzymatic passage in between (5-6 days). Subsequently, the cells were cultured in the same conditions but without the supplementation with 10 µM of SB431542. At passage three, the cells were seeded at 1:1 ratio in non-coated 6-well plates. At passage four, cells were transferred at 1:1.5 ratio to non-coated 175 cm^2 tissue culture treated flasks.

2.2. Directed differentiation protocol through the Lateral Plate Mesoderm

A previously described protocol for directing differentiation via the LPM pathway in human cells [3] was tested as exactly reported using eqiPSCs. These eqiPSCs were unable to navigate the differentiation process and died very soon during the first stages of induction, so no further data could be recorded.

3. Results

Protocols 1 and 2 did not yield MSC-like cells from the eqiPSC line tested in this study. Both protocols were based on spontaneous differentiation and essentially consisted of directly switching the eqiPSC culture media by MSC culture/induction media. In the first place, we tried the Protocol 1 as it is described in the bibliography [1], by seeding eqiPSCs onto vitronectin. However, eqiPSCs did not adapt properly to feeder-free culture and could only be maintained for a few passages with progressive decline in vitality. Therefore, we tried again this protocol but initially seeding eqiPSCs onto iMEFs, which is their regular culture system. Nevertheless, eqiPSCs progressively died after some passages. Subsequently, Protocol 2 was tested, which was very similar to Protocol 1 but included specific small molecules in the media [2]. This protocol neither seemed to work to obtain MSC-like cells from eqiPSCs in our conditions.

For both Protocol 1 and 2, one month after switching culture conditions of the eqiPSCs, the cells changed their morphology towards a rhomboidal shape but did not reach the typical fibroblast-like morphology, and kept growing forming colony-like clusters. Moreover, the cells reduced their ability to adhere to plastic (Figure S1).

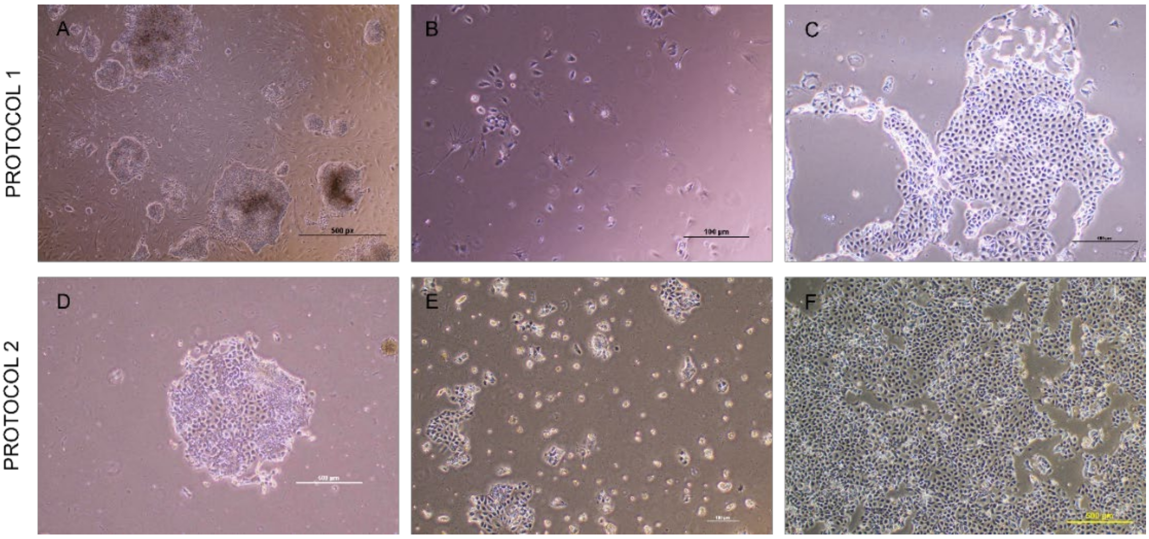

Figure S1. Top row presents equine induced pluripotent stem cells (eqiPSCs) line FD6 after several passages undergoing Protocol 1 differentiation: A) passage 0 (P0), day 3; B): P2, day 10; C) P3, day 19. Bottom row represents eqiPSCs line FD6 after several passages undergoing Protocol 2 differentiation: D) P2, day 15; E) P3, day 20; F) P5, day 29. Scale bars: A, D, F = 500 µm B, C, E = 100 µm.

4. References

[1] M. J. Chung *et al.*, “Differentiation of equine induced pluripotent stem cells into mesenchymal lineage for therapeutic use,” *Cell Cycle*, vol. 18, no. 21, pp. 2954–2971, 2019, doi: 10.1080/15384101.2019.1664224.

[2] S. Lepage, K. Nagy, H.-K. Sung, R. A. Kandel, A. Nagy, and T. G. Koch, “Generation, Characterization, and Multilineage Potency of Mesenchymal-Like Progenitors Derived from Equine Induced Pluripotent Stem Cells,” *Stem Cells Dev.*, vol. 25, no. 1, pp. 80–89, 2016.

[3] Y. Wei *et al.*, “Lateral Mesoderm-Derived Mesenchymal Stem Cells With Robust Osteochondrogenic Potential and Hematopoiesis-Supporting Ability,” *Front. Mol. Biosci.*, vol. 9, no. April, pp. 1–15, 2022, doi: 10.3389/fmolb.2022.767536.

[4] L. Barrachina *et al.*, “Generation of equine induced pluripotent stem cells from cells of embryonic , perinatal and adult tissues,” vol. 1, pp. 1–18, 2025.
