# Supplementary material for: Derivation of Equine Mesenchymal Stem/Stromal Cells from Induced Pluripotent Stem Cells via the Neural Crest Pathway and Characterisation by Immunophenotype and Tri-Lineage Differentiation
Source: Animals (Basel). 2026 May 26;16(11):1618. doi: 10.3390/ani16111618 (PMC13255804; doi:10.3390/ani16111618)
Supplement: Supplementary file 1 [file animals-16-01618-s001.zip › Supplementary Material S2.pdf]

Supplementary Material S2: Representative gating plots for the analysis of CD90 and CD105.

The surface expression of several markers was assessed in the eqiMSCs (equine mesenchymal stem/stromal cells derived from induced pluripotent stem cells) generated in this study. The gating strategy to determine the percentage of positive cells for each marker was previously described by our group [1]. Briefly, dead cells and doubles were excluded, and the positive cells gate was set using unstained controls. Two of the mesenchymal markers, CD90 and CD105, showed lower and more variable expression in eqiMSCs: only eqiMSC lines FD6 and FD8.1 expressed the positive marker CD90 and only eqiMSC line FD8.1 expressed the positive marker CD105. To better represent these results, Figure S2 contains representative gating plots to show how CD90+ and CD105+ cells were gated using unstained controls. The line D1 of bone marrow derived MSCs (eqBM-MSCS) is included for reference (Figure S2.A) and eqiMSC lines FD6, FD7, FD8.1 and FD8.6 are presented in Figure S2.B, Figure S2.C, Figure S2.D and Figure S2.E, respectively.

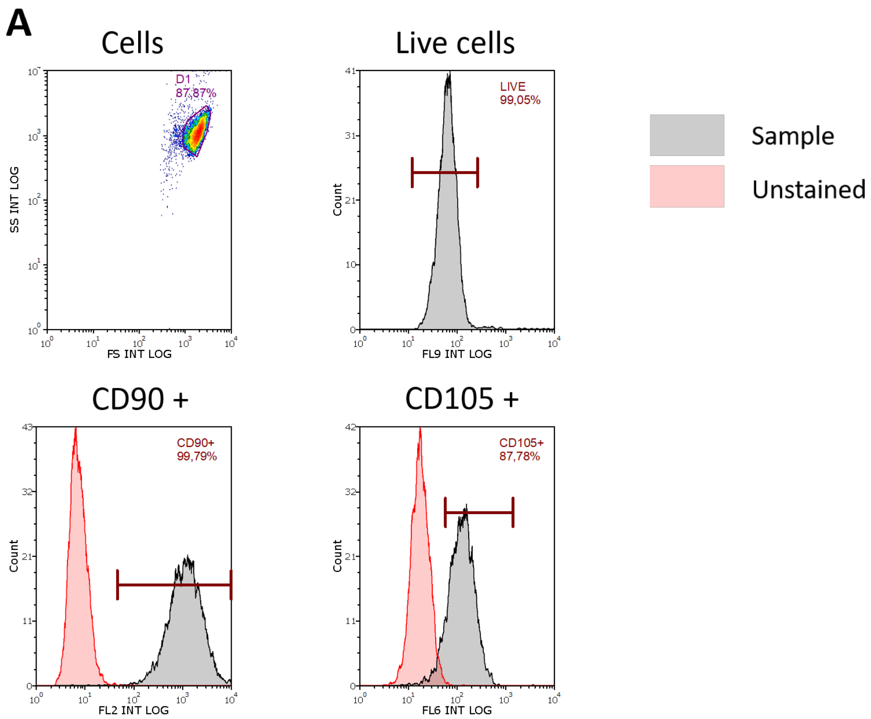

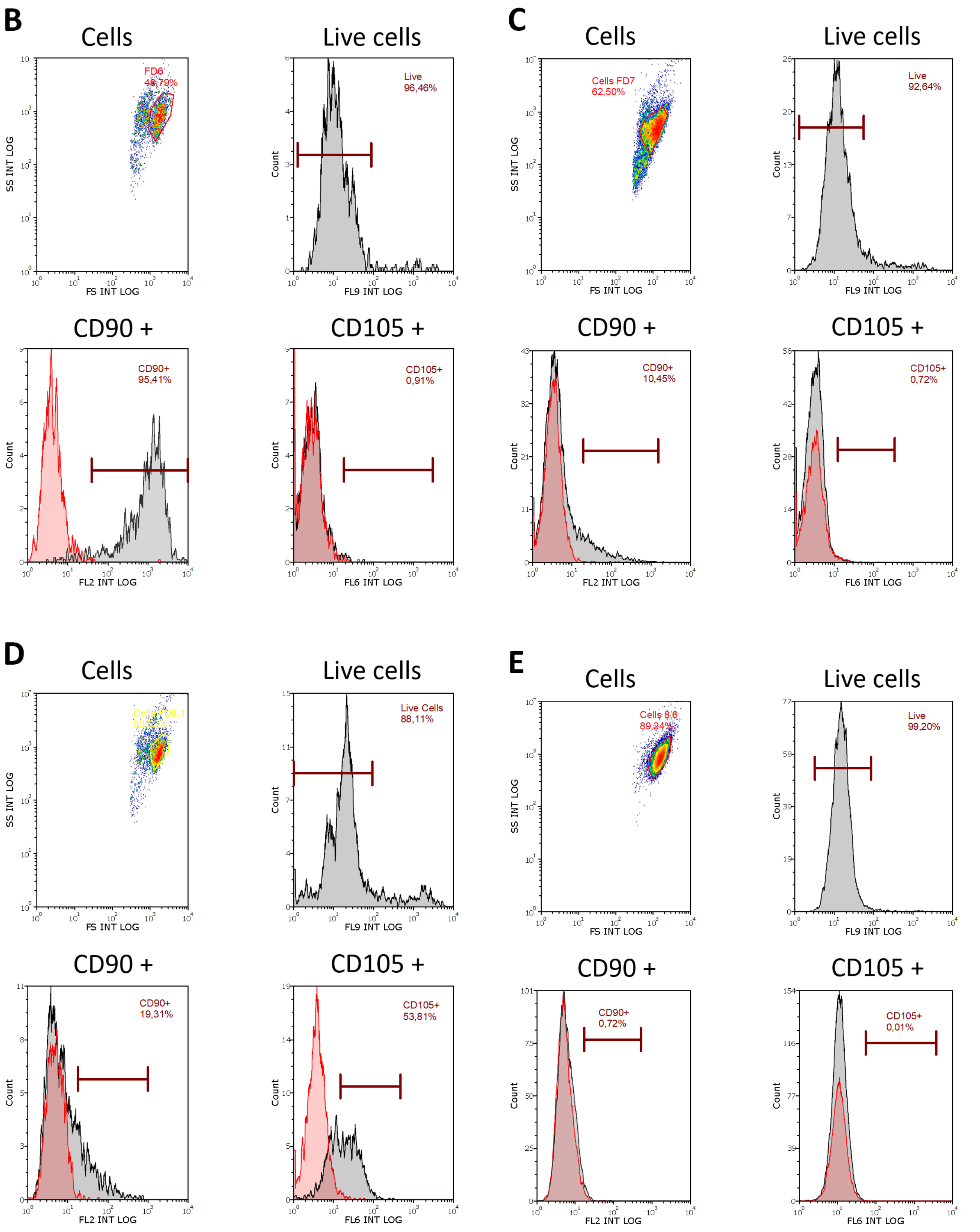

Figure S2. Flow cytometry gating strategy. First row of each group of plots shows how the population of mesenchymal stem/stromal cells (MSCs) was gated in the forward and side scatter (FSC x SSC) plot, dead cells (FSC x FL9) were subsequently excluded. Second row represents CD90+ (FL2) and CD105+ (FL6) cells gated (grey) using unstained control to set the negative population (red). **A,** equine bone marrow-derived MSCs from donor 1 (D1), for reference of positive cell; **B,** equine induced MSCs (eqiMSCs) line FD6; **C**, eqiMSC line FD7; **D**, eqiMSC line FD8.1 and **E**, eqiMSC line FD8.6.

References

[1] A. Cequier *et al.*, "Equine Mesenchymal Stem Cells Influence the Proliferative Response of Lymphocytes: Effect of Inflammation, Differentiation and MHC-Compatibility," *Animals*, vol. 12, no. 8, 2022, doi: 10.3390/ani12080984.
